# People's interest in brain health testing: Findings from an international, online cross-sectional survey

**DOI:** 10.3389/fpubh.2022.998302

**Published:** 2022-10-20

**Authors:** Rebecca B. Carver, Nanna Alida Grit Fredheim, Athanasia Monika Mowinckel, Klaus P. Ebmeier, Barbara Bodorkos Friedman, Tor Atle Rosness, Christian A. Drevon, Sana Suri, William F. C. Baaré, Eniko Zsoldos, Cristina Solé-Padullés, David Bartrés-Faz, Paolo Ghisletta, Laura Nawijn, Sandra Düzel, Kathrine Skak Madsen, Anders M. Fjell, Ulman Lindenberger, Kristine B. Walhovd, Isabelle Budin-Ljøsne

**Affiliations:** ^1^Department of Communication, Norwegian Institute of Public Health, Oslo, Norway; ^2^School of Communication, Leadership and Marketing, Kristiania University College, Oslo, Norway; ^3^Department of Psychology, Center for Lifespan Changes in Brain and Cognition, University of Oslo, Oslo, Norway; ^4^Department of Psychiatry, University of Oxford, Oxford, United Kingdom; ^5^Reviews and Health Technology Assessments Cluster, Norwegian Institute of Public Health, Oslo, Norway; ^6^Department Nutrition, Faculty of Medicine, Institute Basic Medical Sciences, University of Oslo, Oslo, Norway; ^7^Vitas AS, Oslo Science Park, Oslo, Norway; ^8^Wellcome Centre for Integrative Neuroimaging, University of Oxford, Oxford, United Kingdom; ^9^Danish Research Centre for Magnetic Resonance, Centre for Functional and Diagnostic Imaging and Research, Copenhagen University Hospital Amager and Hvidovre, Copenhagen, Denmark; ^10^Department of Medicine, Faculty of Medicine and Health Sciences & Institute of Neurosciences, University of Barcelona, Barcelona, Spain; ^11^Institut d'Investigacions Biomèdiques August Pi i Sunyer (IDIBAPS), Barcelona, Spain; ^12^Faculty of Psychology and Educational Sciences, University of Geneva, Geneva, Switzerland; ^13^UniDistance Suisse, Brig, Switzerland; ^14^Swiss National Centre of Competence in Research LIVES, University of Geneva, Geneva, Switzerland; ^15^Department of Psychiatry, Amsterdam Neuroscience, Vrije Universiteit Amsterdam, Amsterdam, Netherlands; ^16^Center for Lifespan Psychology, Max Planck Institute for Human Development, Berlin, Germany; ^17^Radiography, Department of Technology, University College Copenhagen, Copenhagen, Denmark; ^18^Center for Lifespan Psychology, Max Planck Institute for Human Development, Berlin, Germany; ^19^Max Planck UCL Centre for Computational Psychiatry and Ageing Research, Berlin, Germany; ^20^Department of Food Safety, Norwegian Institute of Public Health, Oslo, Norway

**Keywords:** public perspectives, public health, brain health, mental health, wellbeing, predictive testing, Alzheimer's disease, survey

## Abstract

Brain health entails mental wellbeing and cognitive health in the absence of brain disorders. The past decade has seen an explosion of tests, cognitive and biological, to predict various brain conditions, such as Alzheimer's Disease. In line with these current developments, we investigated people's willingness and reasons to—or not to—take a hypothetical brain health test to learn about risk of developing a brain disease, in a cross-sectional multilanguage online survey. The survey was part of the Global Brain Health Survey, open to the public from 4th June 2019 to 31st August 2020. Respondents were largely recruited via European brain councils and research organizations. 27,590 people responded aged 18 years or older and were predominantly women (71%), middle-aged or older (>40 years; 83%), and highly educated (69%). Responses were analyzed to explore the relationship between demographic variables and responses.

**Results:**

We found high public interest in brain health testing: over 91% would definitely or probably take a brain health test and 86% would do so even if it gave information about a disease that cannot be treated or prevented. The main reason for taking a test was the ability to respond if one was found to be at risk of brain disease, such as changing lifestyle, seeking counseling or starting treatment. Higher interest in brain health testing was found in men, respondents with lower education levels and those with poor self-reported cognitive health.

**Conclusion:**

High public interest in brain health and brain health testing in certain segments of society, coupled with an increase of commercial tests entering the market, is likely to put pressure on public health systems to inform the public about brain health testing in years to come.

## Introduction

The concept of brain health has emerged in recent years to describe the state of brain functioning. It is a multifaceted concept because it refers to how well a person's brain functions across several areas including cognitive, emotional, sensory and motor function (1). The World Health Organization emphasize the importance of brain health to allow a person to realize their full potential over the life course, irrespective of the presence or absence of disorders (2). Others define brain health at any given age as the preservation of optimal brain integrity and mental and cognitive function in the absence of overt brain diseases (3). Recently, a new definition of brain health also takes into consideration mental health, wellbeing, and happiness, defining brain health in adults as “a state of complete physical, mental, and social wellbeing through the continuous development and exercise of the brain” (4). In this study, we provided participants with the following description of brain health, based on the US National Institute on Aging's information to the public (1): “Brain health is about your ability to remember, learn, plan, concentrate, and handle challenges. It is also about your ability to be mentally and emotionally in balance. Simply said, brain health is about making the most of your brain and taking care of it.”

Brain health can be affected by a wide range of brain disorders i.e., neurological and psychiatric disorders, such as dementia, Parkinson, stroke, depression, schizophrenia and autism. Due to increased longevity, brain health related diseases are expected to increase in the coming decades, worldwide (5). Most brain diseases have a multifactorial origin, where genetic and environmental risk factors play an important role. For instance, about 40 percent of dementia cases might be prevented through lifestyle changes, potentially reducing health care needs over the next decades (6). Public awareness of brain health and the associated life factors is therefore becoming an increasingly important public health issue (7). In this paper we argue that public interest in brain health and brain health testing, coupled with a commercial drive for more brain health tests, is likely to put pressure on public health systems in years to come.

There are relatively few studies based on the broad concept of brain health; research is still mostly focused on one or few specific aspects of it (for e.g., dementia, cognition etc.). Studies that have explored brain health awareness find that people are generally conscious of their brain health and are interested in learning more about it, although they are less aware of brain health than other health issues (8–12). Studies report a varying level of knowledge of lifestyle factors (such as sleep, diet, physical activity, substance use etc.) influencing brain health. Studies find low awareness of the importance of systemic diseases, such as cardiovascular disease and diabetes, for the brain (12, 13). Confusion about which activities and factors benefit brain health has been apparent and illustrates the need for more evidence-based information regarding risk-reducing strategies. Although studies find people have positive intentions to change current brain health behavior, the intention-behavior gap is still high in the field of brain health as well (14, 15). Symptoms of cognitive or mental decline, knowledge of disease risk or having family members with brain diseases are reported as some of the key motivating factors for behavioral changes (11, 15).

In line with the numerous studies that have documented high public interest in medical testing (16), several studies have found relatively high public interest in testing for specific brain diseases, such as Alzheimer's disease (AD) (13, 17–20). Public interest in the early detection of dementia seems to be connected with large expectations about the effectiveness of prevention. These expectations may be partly driven by the positive media reporting of medical breakthroughs in general, particularly related to genetic research (21). New genetic tests for assessing risk for specific brain diseases, such as Alzheimer's disease, are becoming available and are often reported in the media (22).

At present however, no single test can comprehensively assess or quantify brain health (3). Different aspects of brain health can be measured using different methods, such as genetic tests, biomarkers, neuroimaging and various cognitive and memory tests (3, 23), but existing tests have varying diagnostic validity, and for many conditions there is often a lack of effective prevention and treatment. Apart from Huntington's Disease and some rare mutations causing early-onset Alzheimer's disease, the predictive value of genetic testing for common brain diseases is uncertain (24), as for instance, many individuals suggested to be at risk for dementia might never develop symptoms, and abnormal disease biomarkers are also prevalent in healthy old people leading to false positives and low specificity of such tests (25). Also, studies typically find only limited influence of genetic information on subsequent illness and risk-related lifestyle changes (26). Consequently, most clinicians do not recommend pre-symptomatic tests for learning about personal risks for dementia, such as Alzheimer's disease (27, 28). Genotyping furthermore raises legal questions about testing protocols, disclosure practices, confidentiality, insurance and employment discrimination, and the availability of follow-up care (19). Nevertheless, the emergence of commercial genetic tests and overly positive media coverage of the benefits of medical testing is rapidly increasing availability and consumer spending on medical testing, including neurological tests (22, 29, 30).

Few studies have explored public interest in undertaking testing to learn about their personal brain health, or what motivates such an interest. Given the substantial public interest in brain health and the growing availability of commercial medical testing, there is a need to explore whether people are interested in testing their brain health, and what motivates such an interest in testing (or not). The purpose of this paper is therefore to explore people's interest in undertaking a hypothetical brain health test to learn about their risk of developing a brain disease.

As part of a large-scale international survey—the Global Brain Health Survey (GBHS) (31)—respondents were asked to imagine “a simple brain health test to learn about risk of developing a brain disease,” With brain health tests, we hence refer to hypothetical, non-invasive tests for risk of non-specified brain disease, rather than any known test available today. The generalized description of the test was intentional to capture overall interest and willingness to learn about the general risk of brain disease, rather than specific diseases. We explored how views differ across individual and sociodemographic characteristics. The survey provided an unusually large international sample of interested people, and, despite the selective nature of the sample, this study provides new knowledge and useful insights about public perspectives on brain health testing that may be relevant for public health policy makers at European and international levels.

## Materials and methods

### The survey

The GBHS was organized as part of the research project “Lifebrain; Healthy minds from 0 to 100 years: Optimizing the use of European brain imaging cohorts,” a 5 $\frac{1}{2}$—year long research project in the Horizon 2020 program of the European Commission (32). The consortium combines data from 11 European cohorts to explore biological, cognitive, environmental, social, occupational, and lifestyle factors affecting brain health. The survey items in this study are a part of the Global Brain Health Survey, which covered several topics related to brain health and was available in 14 languages. The survey was anonymous and open to anyone above the age of 18 years consenting to participate. The whole survey took 15–20 min to complete and was freely available on the website www.lifebrain.uio.no. There was no compensation and participants had to have their own internet access, so incentives for fraud or duplications were low. A comprehensive description of the survey and its design can be found elsewhere (31). For this study, we investigated respondents' answers to six of the questions in the Global Brain Health Survey that were related to the theme of brain health testing. These were: (1) respondents' willingness to undertake such a test for their brain health to reveal risk of developing a brain disease, and (2) even if such diseases were unpreventable or not treatable, (3) reasons why they would take or (4) not take a brain health test, (5) their likely reactions to brain health test results and (6) the criteria they considered important, such as tests being affordable, quick, accurate, or painless.

### Sampling

The original objective of the survey was to reach as many people as possible in Europe and beyond, and the goal was to achieve a sample size of 10,000 (31). To reach this large number, a convenient sampling strategy was adopted and the survey was distributed using using newsletters, information on websites and social media of the participating brain health organizations and research networks in the Lifebrain project. This included the brain research registries Hersenonderzoek in the Netherlands and Join Dementia Research in the UK with a volunteer base of 34,000+ and 50,000+ respectively, of which ~20% participated in this survey. The large proportion of participants recruited via such registers makes it likely that the overall sample was particularly interested in brain health and thus not representative of the general population. The remainder of the respondents in the UK and NL had been recruited through research organizations, brain foundations and research networks connected to the Lifebrain project, like the participants in the other countries. Data was collected between June 2019 to August 2020. A full description of the population and sampling strategy has been published previously (12).

### Measures

The GBHS survey included 16 multiple choice questions that covered four themes: perceptions of brain health, interest in brain health tests, motivations to look after one's brain health and support needed to promote brain health. In this paper we investigate the responses to the part of the survey that addressed respondents' interest in undertaking brain health tests, defined as a willingness to test for risk of developing brain disease. These were:

1) Willingness to take a brain health test: “Imagine a simple brain health test to learn about risk of developing a brain disease. Would you wish to take such a test?” Respondents could select: Yes—definitely; Yes—probably; No—probably not; No—definitely not.

2) Willingness to test for unpreventable or untreatable diseases: “Would you take a test even if it provides information about a disease that cannot be prevented or treated?” Respondents could choose between: Yes—definitely; Yes—probably; No—probably not; No—definitely not.

3) Reasons for taking a brain health test: “Why would you take a brain health test?” Respondents were asked to select the one or two most important out of the following: (a) To get information about my cognitive and mental health, (b) To determine my risk of developing a brain disease, (c) To respond if I am at risk, e.g., change my lifestyle, seek counseling, or start treatment, (d) To prepare myself for the future (e.g., inform my family about the risk), (e) Other motivation (please specify).

4) Reasons for NOT wanting to take a brain health test, if they answered No to question 1; “Would you wish to take a test”): “Why would you NOT take a brain health test¿‘ Respondents were asked to select up to two most important reasons, out of the following options: (a) I do not want to worry about something that may not happen, (b) I do not want to know about a disease that could not be prevented or treated, (c) I would be frightened by the result, (d) There is nothing I can do for my brain health anyway, or (e) Other reasons (please specify).

5) Likely reactions to test results on brain health risk: “Imagine you undergo a brain health test, and it shows that you have a risk of developing brain disease. What would be your most likely reaction?” Respondents were presented with a list of reactions and were asked to rate these using a four-item Likert scale (definitely yes, fairly likely, fairly unlikely, definitely not): (a) I would seek professional help (e.g., my doctor), (b) I would seek advice from family and friends, (c) I would seek information online/at the library, (d) I would change my lifestyle if required, (e) I would plan for the future, and (f) Is there anything else you think you might do? Please describe (free text).

6) Brain health test criteria: Respondents were asked to imagine it was possible to take a simple brain health test, like measuring blood pressure or cholesterol levels, to reveal risk of developing brain disease. Respondents were asked to select the one to three most important characteristics that such a brain health test should have: (a) Affordable, (b) Quick to take, (c) Accurate, (d) Painless, (e) Subsidized by social security (via the GP), (f) Offered online with direct access to the results, (g) Other (please specify).

We also explore 10 demographic variables related to: age, gender (self-identified), education level, relationship status, experience or education in health care, experience of long-standing illness or disability, experience of taking care of a family member with brain disease, experience with taking part in brain research, self-assessed cognitive health, and self-assessed mental health. For the variable “self-assessed cognitive health” respondents were asked: “How would you describe your ability to think, remember and learn? (Excellent, above average, average, below average or very poor). For “self-assessed mental health” respondents were asked; “How would you describe your ability to balance your mood and emotional well-being?” (Excellent, above average, average, below average or very poor). For the gender category, there were four options: Male, female, other, prefer not to answer.

### Analysis

For the analyses of demographic differences, responses were analyzed using generalized binomial linear models with R version 4.1.0 (33) at a 99% level of significance. To reduce unnecessary complexity, survey questions which contained data from multiple response categories, were collapsed into binary response categories. For the first question (Would you wish to take such a test?) and second question (*Would you take a test even if the disease cannot be treated or prevented*?), the responses “Yes, definitely” and “yes, probably” were categorized as a positive association between the question and the response, while “No, probably not,” and “No, definitely not” were categorized as a negative association. Responses for the third, fourth and sixth questions were binary. For the fifth question (*What would be your most likely reaction?)*, the response categories “Definitely yes” and “Fairly likely” were categorized as being likely to react in the given way (positive association), and “Fairly unlikely” and “Definitely not” were categorized as being unlikely to react in the given way (negative association).

For each response we estimated 10 models, using a single predicting demographic variable for each model, to analyse the relationship between responses and demographic characteristics. To simplify data interpretation, complex demographic variables were reduced to three or fewer data categories. Education level was reduced to either “higher education” (university/college degree) or “lower education” (primary school, special educational school, secondary school, vocational training). Age was reduced to three categories, “young adult” (40 and below), “middle-aged” (41 to 60 years) or “old adult” (above 60 years). Self-assessed mental and cognitive health were categorized as either “good” mental or cognitive health (encompassing response categories “average,” “above average” and “excellent”), or “poor” mental or cognitive health (below average or very poor). Relationship status was reduced as either in a stable relationship (“married,” “stable relationship, not married”) or single (“divorced/separated,” “widow/widower,” “single”). The response category with the highest number of data points was used as the reference group.

Due to the large sample size, almost all group differences were statistically significant but not necessarily of practical importance. Thus, in the results section we report only Odds Ratios (ORs) of the binarized responses showing the most important differences. All reported ORs in the text are significant at the 1% level of probability. Complete tables of all the descriptive statistics for all variables are provided in the online Supplementary material, as well as models for continuous data for robustness.

## Results

### Respondent characteristics

Twenty seven thousand five hundred and ninety people from 81 countries participated in the survey, mostly from Europe (98%): 36.8% in the United Kingdom, 25.5% in the Netherlands, 12.9% in Norway, 7.6% in Spain, 4.0% in Denmark, 3.8% in Germany, 2.8% in Sweden, 1.1% in Italy and 1.1% in Ukraine. Respondents outside of Europe primarily lived in the United States (0.6%) or Turkey (0.5%). 1.7% were from other parts of the world. Most respondents were middle aged-or older (>40 years; 83.6%), female (71.1%), had a university/college degree (68.6%), and were in a stable relationship (71.8%). Forty-three percent of the respondents had participated in brain research, 38.5% had education or work experience in health care, 46.5% had experience with looking after a family member with brain disease and 40.4% had long-standing illness, disability, or health problems.

**Figure 1:**
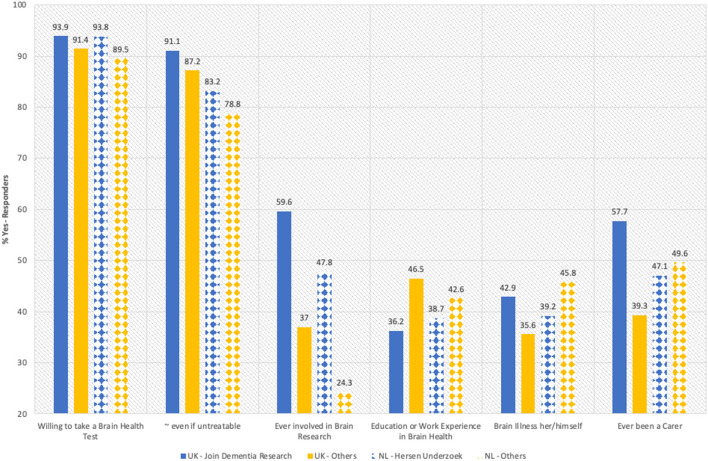
Comparison between participants from research registries and the remainder from same country. The graph shows how the participants recruited from research registries *Join Dementia Research* and *Hersenonderzoek* answered relevant questions compared with other respondents from the same country (UK and NL others).

Over half (58%) had been recruited through the two research registries in the UK and the Netherlands. To illustrate the selectivity of recruitment, we compared those participants recruited through *Join Dementia Research* (*n* = 9,878) in the UK and *Hersenonderzoek* (*n* = 6,117) in the Netherlands (NL) with the remainder of respondents from UK (*n* = 1,074) and NL (*n* = 974), respectively. Figure 1 shows that registry members were slightly more willing to take a ‘brain health test' than others. As expected, a larger proportion of registry participants had taken part in brain research than respondents not on a registry. Fewer had education or work experience in brain health, thus highlighting a separate source of interest for non-registry members. UK but not Dutch registry participants were more commonly looking after a family member (“ever been a carer”; 57.7% vs. 39.3%). This illustrates not only the obvious differences, but also the selectiveness of the respondents not part of research registries (the percentage of other respondents engaged in research was >25%).

### Willingness to take a brain health test

Most respondents (60%) would “definitely” take a simple brain health test to learn about risk of developing a brain disease, whereas 31% would “probably” take such a test. Only 1% would “definitely not” take a test.

Table 1 shows the differences in willingness to take a brain health test, as well as tests for untreatable diseases, between demographic groups of respondents. Respondents willing to take a test had higher chances of being men (OR 1.75, 99% CI 1.52–2.01), having lower education (OR 1.52, 99% CI 1.34–1.72), and poor self-reported cognitive health (OR 1.48, 99% CI 1.13–1.93). They were also more likely to have had experience with long-standing illness or disability (OR 1.25, 99% CI 1.11–1.40) and to have had participated in brain health research (OR 1.31, 99% CI 1.17–1.46).

**Table 1:** Probability of taking a brain health test (question 1), and probability of taking a test even if it provides information about a disease that cannot be prevented or treated (question 2), by demographic groups.

**Variable**	**Characteristics**	**Willingness to take a brain health test**			~**even if illness is not preventable or untreatable**		
		**%**	**OR**	**99% CI**	**%**	**OR**	**99%CI**
Gender	Women	90.0			84.5		
	Men	94.0	1.75	1.52–2.01	90.5	1.76	1.56–1.97
	Other/Undisclosed	80.9	0.47	0.27–0.84	85.8	1.11	0.54–2.29
Age	>60 years	92.6			88.4		
	41–60 years	90.2	0.73	0.65–0.83	85.4	0.77	0.69–0.85
	≤ 40 years	89.0	0.65	0.56–0.76	81.9	0.59	0.52–0.67
Education	Higher education	90.1			85.1		
	Lower education	93.2	1.52	1.34–1.72	88.8	1.39	1.25–1.55
Cognitive health	Average or above	90.9			85.9		
	Below average	93.7	1.48	1.13–1.93	91.8	1.85	1.45–2.36
Mental health	Average or above	91.1			86.1		
	Below average	91.1	1.00	0.85–1.17	87.3	1.11	0.96–1.28
Caregiver exp.	No	90.8			85.3		
	Yes	91.4	1.09	0.97–1.21	87.4	1.20	1.09–1.32
Illness experience	No	90.4			84.7		
	Yes	92.1	1.25	1.11–1.40	88.5	1.39	1.26–1.54
Brain research exp.	No	90.2			84.6		
	Yes	92.3	1.31	1.17–1.46	88.4	1.39	1.26–1.54
Health care exp.	No	92.2			87.9		
	Yes	89.3	0.70	0.63–0.78	83.6	0.71	0.64–0.78

% Indicates the proportion of participants answering “Yes, definitely” or “yes, probably” to the question, with the remainder of participants answering “No—probably not” or “No—definitely not.” OR, Odds ratio; CI, Confidence interval.

Young respondents (40 or younger) were less likely to want to take a brain health test (OR 0.65, 99% CI 0.56–0.76), as well as respondents with employment and/or education in healthcare (OR 0.70, 99% CI 0.63–0.78).

### Willingness to test for unpreventable or untreatable diseases

When asked whether they would want to test for risk of developing brain disease that was unpreventable or untreatable, 43% of respondents would “definitely” take a brain health test even if it provided such information, and 43% would “probably” do the same.

Table 1 shows that respondents with lower education (OR 1.39, 99% CI 1.25–1.55), poor self-reported cognitive health (OR 1.85, 99% CI 1.45–2.36), personal experience of chronic illness (OR 1.39, 99% CI 1.26–1.54), or who had cared for a family member with brain disease (OR 1.20, 99% CI 1.09–1.32) were more likely to take a brain health test even for an untreatable disease than respondents without these characteristics. Furthermore, men were more likely to take such a test compared to women (OR 1.76 99% CI 1.56–1.97).

Young respondents (40 and below) (OR 0.59, 99% CI 0.52–0.67) and respondents with employment and/or education within healthcare were less likely to test for risk of disease that is unpreventable and untreatable (OR 0.71, 99% CI 0.64–0.78) compared to older respondents (above 60) and those without healthcare experience.

### Reasons for taking a brain health test

**Figure 2:**
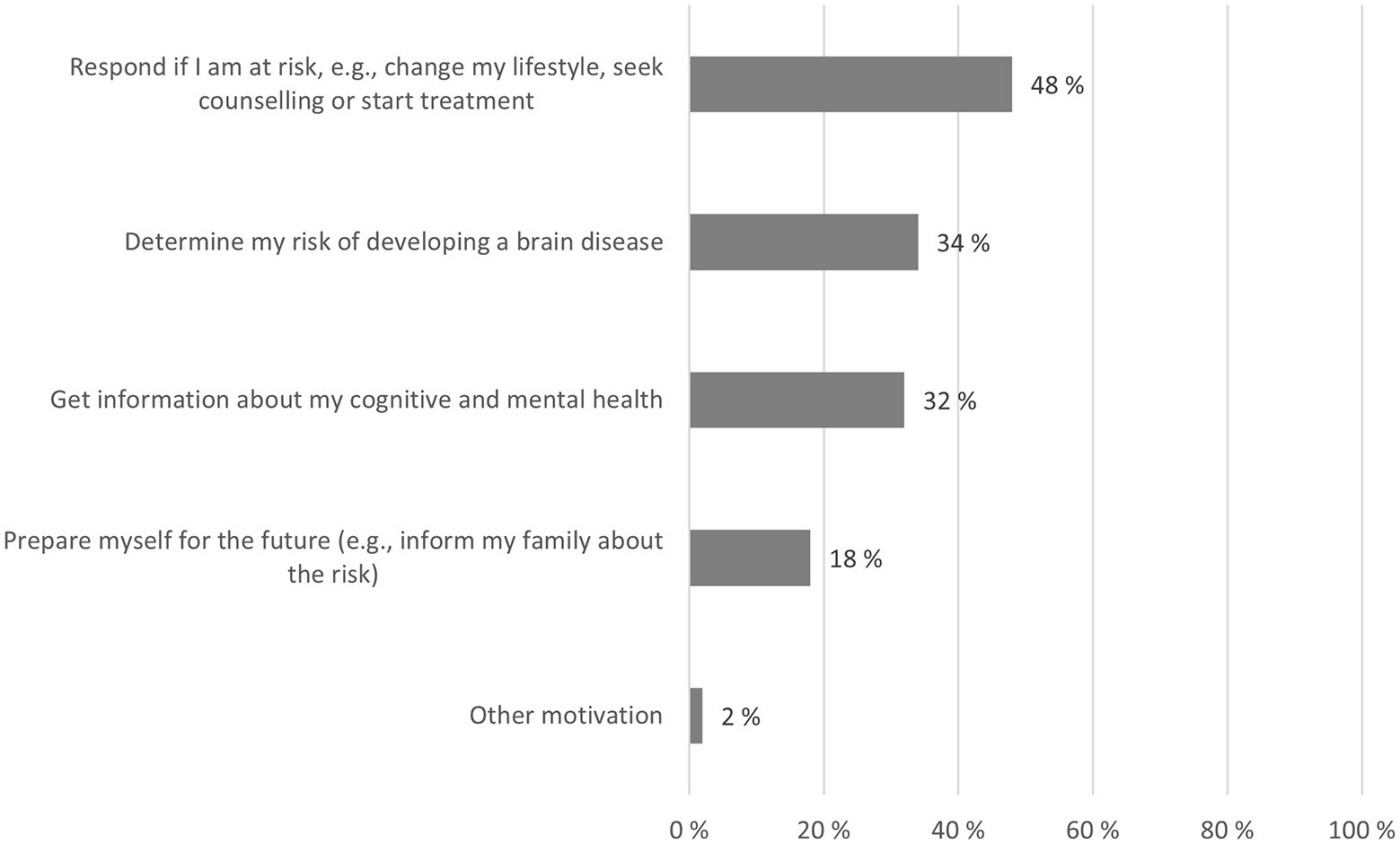
Reasons for taking a brain health test. Respondents who answered they would take a brain health test could select up to two most important reasons for doing so. Percentages indicate the proportion of respondents who chose each reason (percentages exceed 100% in total because respondents could choose up to two reasons).

As shown in Figure 2, the main reason (48%) for wanting to take a brain health test was to be able to respond if found to be at risk of a brain disease. The other two main reasons were learning about the risk of developing a brain disease (34%) and to get information about cognitive and mental health (32%).

Table 2 shows that, compared to older respondents, younger respondents were more likely to take a test in order to respond if they were at risk (OR 1.50, 99% CI 1.37–1.66), and to get information about their cognitive and mental health (OR 1.74, 99% CI 1.58–1.91), but were less likely to take a test to (just) learn about their risk of developing a brain disease (OR 0.75, 95% CI 0.68–0.83).

**Table 2:** Reasons for wanting to take a brain health test.

**Variable**	**Characteristics**	**Respond if at risk**			**Learn about risk**			**Get information**		
		**%**	**OR**	**99% CI**	**%**	**OR**	**99%CI**	**%**	**OR**	**99%CI**
Gender	Women	47.8			33.8			31.0		
	Men	49.4	0.98	0.91–1.05	34.8	0.98	0.91–1.05	35.5	1.16	1.07–1.25
	Other/Undisclosed	44.3	1.07	0.64–1.77	20.6	0.57	0.32–1.01	36.6	1.58	0.95–2.61
Age	>60 years	46.2			35.8			31.2		
	41–60 years	48.4	1.16	1.08–1.25	34.0	0.96	0.89–1.03	29.6	0.96	0.89–1.04
	≤ 40 years	53.4	1.50	1.37–1.66	28.7	0.75	0.68–0.83	41.7	1.74	1.58–1.91
Education	Higher education	49.5			32.6			32.5		
	Lower education	45.3	0.77	0.72–0.83	36.9	1.15	1.07–1.24	31.8	0.92	0.85–0.99
Cognitive health	Average or above	48.5			33.9			32.1		
	Below average	44.0	0.78	0.68–0.89	35.0	1.00	0.87–1.15	34.4	1.06	0.92–1.22
Mental health	Average or above	48.2			34.4			31.7		
	Below average	48.4	1.01	0.92–1.11	31.4	0.87	0.78–0.96	36.3	1.25	1.13–1.38
Caregiver exp.	No	49.8			32.5			35.4		
	Yes	46.3	0.84	0.79–0.90	35.7	1.15	1.07–1.23	28.7	0.72	0.67–0.77
Illness experience	No	48.5			34.2			31.5		
	Yes	47.7	0.93	0.87–0.99	33.7	0.95	0.89–1.02	33.4	1.06	0.99–1.13
Brain research exp.	No	48.2			33.3			33.4		
	Yes	48.1	0.95	0.89–1.01	34.9	1.04	0.97–1.11	30.8	0.85	0.79–0.91
Health care exp.	No	48.4			34.5			32.7		
	Yes	47.8	1.04	0.98–1.12	33.1	0.99	0.92–1.06	31.6	1.00	0.93–1.07
**Variable**	**Characteristics**	**Prepare for the future**			**Other motivation**			
		**%**	**OR**	**99% CI**	**%**	**OR**	**99% CI**	**%**	**OR**	**99%CI**
Gender	Women	18.4			2.0					
	Men	18.3	0.94	0.86–1.03	2.0	0.93	0.73–1.19			
	Other/Undisclosed	22.9	1.53	0.88–2.68	3.1	1.71	0.45–6.39			
Age	>60 years	21.7			2.0					
	41–60 years	17.1	0.76	0.70–0.83	2.3	1.20	0.95–1.52			
	≤ 40 years	12.0	0.51	0.45–0.58	1.4	0.76	0.53–1.09			
Education	Higher education	18.6			1.8					
	Lower education	18.1	0.93	0.85–1.01	2.6	1.42	1.13–1.78			
Cognitive health	Average or above	18.3			1.9					
	Below average	19.7	1.05	0.89–1.24	4.2	2.20	1.57–3.09			
Mental health	Average or above	18.7			1.8					
	Below average	16.6	0.86	0.76–0.98	3.5	2.00	1.53–2.60			
Caregiver exp.	No	15.8			1.6					
	Yes	21.5	1.46	1.35–1.58	2.5	1.63	1.30–2.04			
Illness experience	No	17.7			1.6					
	Yes	19.4	1.09	1.01–1.19	2.7	1.71	1.37–2.14			
Brain research exp.	No	15.5			1.9					
	Yes	22.2	1.53	1.41–1.66	2.2	1.12	0.90–1.40			
Health care exp.	No	18.7			2.2					
	Yes	18.0	1.00	0.92–1.08	1.7	0.81	0.64–1.03			

% Indicates the proportion of participants selecting each reason. OR, Odds ratio; CI, Confidence interval.

Respondents with poor cognitive health (OR 0.78, 99% CI 0.68–0.89) and those with lower education (OR 0.77, 95% CI 0.72–0.83) were less likely to take a test to respond to risk compared to respondents without these characteristics.

Respondents who wanted to take a test to get information about cognitive and mental health were more likely male (OR 1.16, 95% CI 1.07–1.25), and with poor mental health (OR 1.25, 99% CI 1.13–1.38).

Respondents more interested in preparing for the future were more likely to have had participated in brain research (OR 1.53, 99% CI 1.41–1.66) and to have had experience in taking care of a family member with brain disease (OR 1.46, 99% CI 1.35–1.58), than respondents without these characteristics. Younger respondents were less likely to take a test in order to prepare for the future (OR 0.51, 99% CI 0.45–0.58).

### Reasons for NOT taking a brain health test

**Figure 3:**
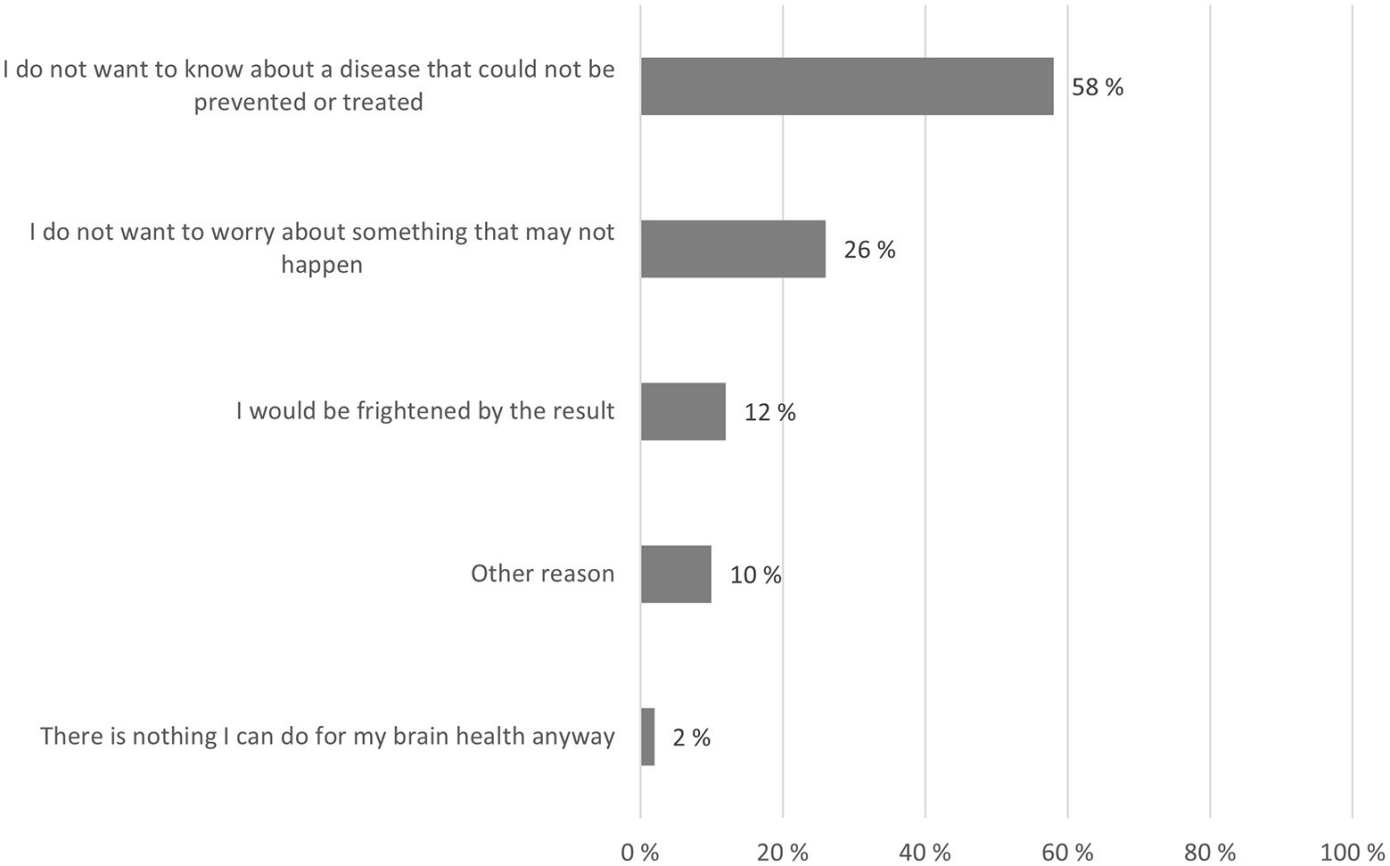
Reasons for NOT taking a brain health test. Respondents who answered they would not take a test, were asked to select up to two most important reasons for not doing so. Numbers indicate the percentage of respondents who chose each reason (percentages exceed 100% in total because respondents could choose up to two reasons).

Of the respondents who did not want to take a test (9%), most did not want to know about a disease that could not be prevented or treated, and a quarter did not want to worry about something that might not happen (Figure 3).

Table 3 shows that, out of the respondents who did not want to take a test, respondents with lower levels of education would to a larger extent not want to worry about something that might not happen (OR 1.57, 99% CI 1.20–2.04), and would be more frightened by the results (OR 1.49, 99% CI 1.04–2.14) compared to those with higher levels of education.

**Table 3:** Reasons for NOT wanting to take a brain health test.

**Variable**	**Characteristics**	**Do not want to know**			**Do not want to worry**			**Frightened by the result**		
		**%**	**OR**	**99% CI**	**%**	**OR**	**99%CI**	**%**	**OR**	**99%CI**
Gender	Women	59.4			26.7			11.7		
	Men	55.0	0.84	0.64–1.09	27.3	1.03	0.76–1.39	9.6	0.80	0.51–1.24
	Other/Undisclosed	32.0	0.32	0.11–0.98	40.0	1.83	0.63–5.29	16	1.43	0.35–5.91
Age	>60 years	60.1			27.5			8.9		
	41–60 years	56.2	0.85	0.67–1.08	26.5	0.95	0.73–1.23	11.8	1.38	0.93–2.03
	≤ 40 years	58.8	0.95	0.71–1.27	26.9	0.97	0.70–1.33	15.4	1.87	1.21–2.89
Education	Higher education	61.0			24.7			10.3		
	Lower education	49.4	0.62	0.49–0.80	34.0	1.57	1.20–2.04	14.7	1.49	1.04–2.14
Cognitive health	Average or above	58.3			27.1			11.3		
	Below average	58.1	0.99	0.59–1.67	23.8	0.84	0.46–1.54	14.3	1.31	0.63–2.75
Mental health	Average or above	58.1			27.2			10.5		
	Below average	59.3	1.05	0.77–1.43	25.6	0.92	0.65–1.31	17.3	1.78	1.17–2.72
Caregiver exp.	No	55.7			28.0			12.5		
	Yes	61.4	1.27	1.02–1.57	25.7	0.89	0.70–1.13	9.9	0.77	0.55–1.08
Illness experience	No	58.8			26.7			10.9		
	Yes	57.2	0.94	0.75–1.17	27.5	1.04	0.82–1.33	12.3	1.15	0.82–1.61
Brain research exp.	No	57.7			29.2			12.8		
	Yes	59.3	1.07	0.86–1.33	23.1	0.73	0.57–0.93	8.9	0.67	0.47–0.95
Health care exp.	No	56.6			27.9			12.2		
	Yes	60.1	1.15	0.93–1.43	25.9	0.90	0.71–1.14	10.4	0.84	0.60–1.17
**Variable**	**Characteristics**	**Other reasons**			**Nothing I can do anyway**			
		**%**	**OR**	**99% CI**	**%**	**OR**	**99% CI**	**%**	**OR**	**99%CI**
Gender	Women	10.2			1.0					
	Men	13.0	1.31	0.88–1.96	3.2	3.39	1.37–8.34			
	Other/Undisclosed	20.0	2.20	0.60–8.09	4.0	4.27	0.29–63.5			
Age	>60 years	9.2			2.4					
	41–60 years	13.4	1.53	1.05–2.22	1.0	0.40	0.15–1.07			
	≤ 40 years	8.9	0.97	0.59–1.59	0.4	0.16	0.02–1.10			
Education	Higher education	10.7			1.0					
	Lower education	11.5	1.08	0.74–1.59	2.9	3.09	1.28–7.45			
Cognitive health	Average or above	10.9			1.3					
	Below average	9.5	0.86	0.36–2.06	4.8	3.87	1.08–13.8			
Mental health	Average or above	11.3			1.4					
	Below average	7.7	0.65	0.37–1.15	1.9	1.37	0.43–4.41			
Caregiver exp.	No	11.4			1.5					
	Yes	10.1	0.87	0.62–1.22	1.3	0.83	0.34–2.02			
Illness experience	No	10.5			1.5					
	Yes	11.4	1.09	0.77–1.54	1.3	0.83	0.32–2.12			
Brain research exp.	No	8.8			1.7					
	Yes	14.3	1.72	1.23–2.41	1.0	0.58	0.21–1.58			
Health care exp.	No	10.6			1.7					
	Yes	11.1	1.05	0.75–1.47	1.1	0.60	0.24–1.51			

% Indicates the proportion of participants selecting each reason. OR, Odds ratio; CI, Confidence interval.

Younger respondents and those with poor mental health would to a larger extent avoid testing because they would be frightened by the results compared to older respondents (OR 1.87, 99% CI 1.21–2.89) and those with good mental health (OR 1.78, 99% CI 1.17–2.72).

Respondents with experience of taking care of a family member with brain disease were more likely to not want to know about a non-preventable or untreatable disease (OR 1.27, 99% CI 1.02–1.57) compared to those without such experience.

### Likely reactions to test results

**Figure 4:**
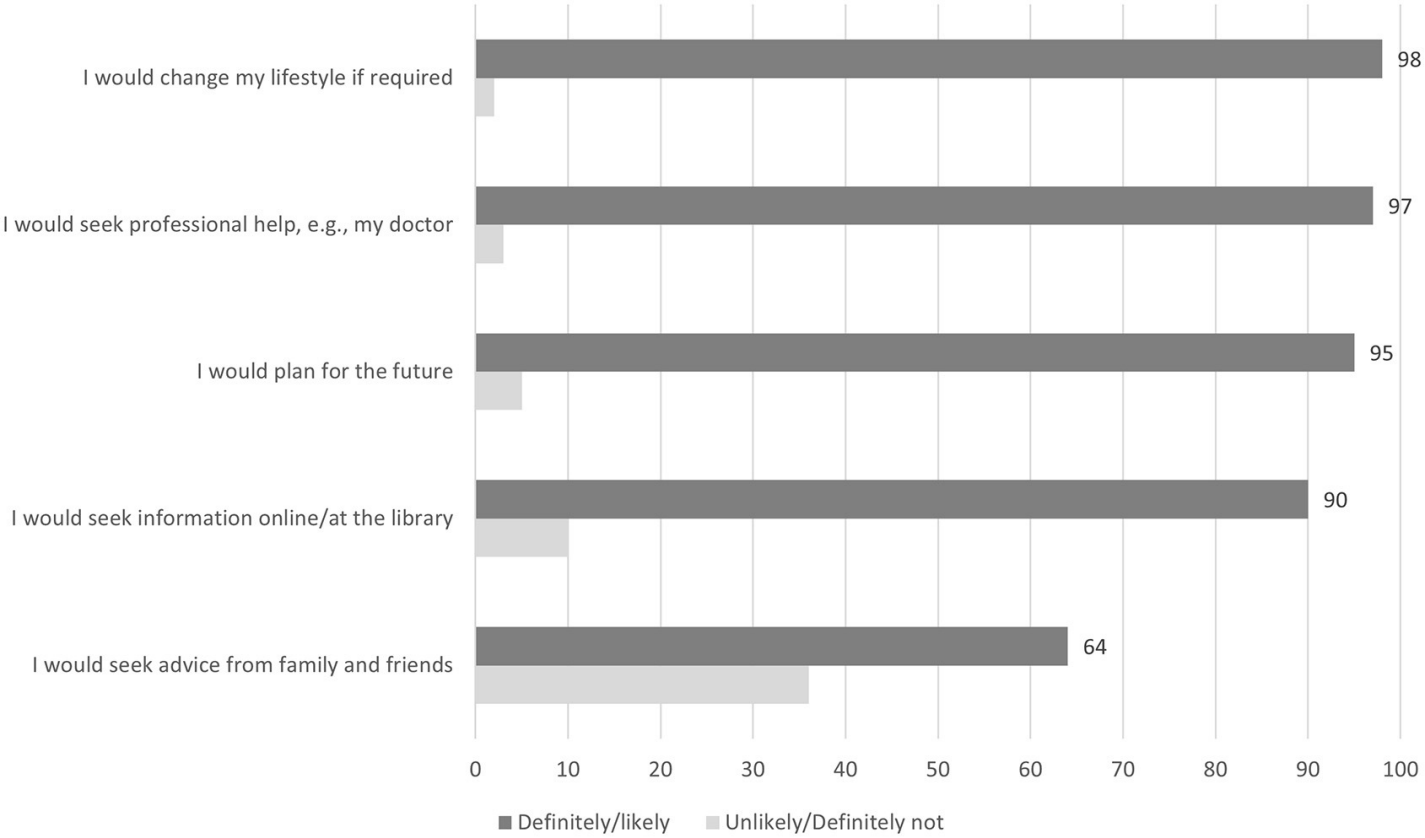
Likely reactions to brain health test results. Numbers indicate the percentage of participants rating the given responses as positive (“Definitely yes” or “Fairly likely”) or negative (“Fairly unlikely” or “Definitely not”).

Almost all respondents (above 95%) said that they would definitely or be fairly likely to change their lifestyle if necessary, seek professional help, and plan for the future based on the results of the (hypothetical) brain health test (Figure 4).

Table 4 shows that young respondents were less likely to seek professional help (OR 0.51, 99% CI 0.40–0.65), or to use test results to plan for the future (OR 0.66, 99% CI 0.55–0.80) compared to older respondents.

**Table 4:** Likely reactions to test results if found to be at risk of brain disease.

**Variable**	**Characteristics**	**Change my lifestyle**			**Seek professional help**			**Plan for the future**		
		**%**	**OR**	**99% CI**	**%**	**OR**	**99%CI**	**%**	**OR**	**99%CI**
Gender	Women	98.0			96.8			95.6		
	Men	97.2	0.71	0.57–0.89	97.3	1.21	0.98–1.50	93.8	0.70	0.60–0.82
	Other/Undisclosed	90.2	0.19	0.08–0.41	91.8	0.37	0.16–0.87	86.1	0.29	0.15–0.57
Age	>60 years	97.7			97.7			95.3		
	41–60 years	98.0	1.16	0.91–1.47	96.6	0.66	0.53–0.81	95.6	1.08	0.91–1.28
	≤ 40 years	97.4	0.89	0.67–1.19	95.6	0.51	0.40–0.65	93.0	0.66	0.55–0.80
Education	Higher education	97.7			96.7			95.7		
	Lower education	97.9	1.07	0.85–1.34	97.5	1.31	1.07–1.61	93.4	0.63	0.55–0.73
Cognitive health	Average or above	97.9			96.9			95.2		
	Below average	95.3	0.43	0.31–0.59	96.8	0.94	0.65–1.37	92.8	0.65	0.50–0.85
Mental health	Average or above	98.0			97.1			95.5		
	Below average	96.0	0.49	0.38–0.63	95.8	0.68	0.53–0.86	92.2	0.56	0.47–0.67
Caregiver exp.	No	97.5			97.0			93.8		
	Yes	98.1	1.34	1.08–1.67	96.8	0.93	0.78–1.12	96.4	1.77	1.52–2.06
Illness experience	No	98.0			96.7			95.3		
	Yes	97.4	0.74	0.60–0.91	97.2	1.19	0.98–1.43	94.6	0.85	0.73–0.98
Brain research exp.	No	97.6			96.9			94.3		
	Yes	98.0	1.25	1.01–1.56	97.0	1.01	0.84–1.22	96.0	1.47	1.26–1.71
Health care exp.	No	97.7			97.5			94.6		
	Yes	97.9	1.09	0.87–1.35	96.1	0.65	0.54–0.77	95.6	1.25	1.07–1.45
**Variable**	**Characteristics**	**Seek information**			**Seek advice**			
		**%**	**OR**	**99% CI**	**%**	**OR**	**99% CI**	**%**	**OR**	**99%CI**
Gender	Women	92.0			65.5					
	Men	86.6	0.57	0.51–0.63	62.1	0.86	0.80–0.93			
	Other/Undisclosed	91.2	0.91	0.40–2.05	62.5	0.88	0.54–1.43			
Age	>60 years	89.5			60.4					
	41–60 years	91.6	1.27	1.13–1.44	65.2	1.23	1.14–1.32			
	≤ 40 years	90.3	1.09	0.93–1.26	73.6	1.82	1.65–2.02			
Education	Higher education	92.3			64.7					
	Lower education	86.4	0.53	0.48–0.59	64.3	0.98	0.91–1.06			
Cognitive health	Average or above	90.5			64.7					
	Below average	89.6	0.90	0.73–1.13	61.6	0.87	0.76–1.01			
Mental health	Average or above	90.3			65.1					
	Below average	91.2	1.11	0.94–1.30	60.7	0.83	0.75–0.91			
Caregiver exp.	No	89.6			63.2					
	Yes	91.4	1.24	1.12–1.39	66.0	1.13	1.06–1.21			
Illness experience	No	90.5			65.3					
	Yes	90.4	1.00	0.90–1.12	63.5	0.92	0.86–0.99			
Brain research exp.	No	90.6			65.7					
	Yes	90.3	0.97	0.87–1.08	63.0	0.89	0.83–0.95			
Health care exp.	No	89.6			64.3					
	Yes	91.8	1.29	1.16–1.45	64.9	1.03	0.96–1.10			

% Indicates the proportion of participants selecting reaction. OR, Odds ratio; CI, Confidence interval.

Respondents with lower education were more likely to seek professional help (OR 1.31, 99% CI 1.07–1.61) but less likely to plan for the future (OR 0.63, 99% CI 0.55–0.73) or seek information (OR 0.53, 99% CI 0.48–0.59) compared to respondents with higher education. In comparison, respondents with employment or education within healthcare were less likely to seek professional help (OR 0.65, 99% CI 0.54–0.77), but more likely to plan for the future (OR 1.25, 99% CI 1.07–1.45) and seek information (OR 1.29, 99% CI 1.16–1.45) compared to respondents without such experience. Respondents who had taken care of next of kin with brain disease were also more likely to plan for the future (OR 1.77, 99% CI 1.52–2.06) and to seek information (OR 1.24, 99% CI 1.12–1.39), in addition to changing their lifestyle (OR 1.34, 99% CI 1.08–1.67), compared to those without such experience.

Respondents with health-issues were overall less interested in changing their lifestyle than other respondents, including those with experience of long-standing disability or illness (OR 0.74, 99% CI 0.60–0.91), with poor cognitive health (OR 0.43, 99% CI 0.31–0.59), or poor self-rated mental health (OR 0.49, 99% CI 0.38–0.63).

### Brain health test criteria

**Figure 5:**
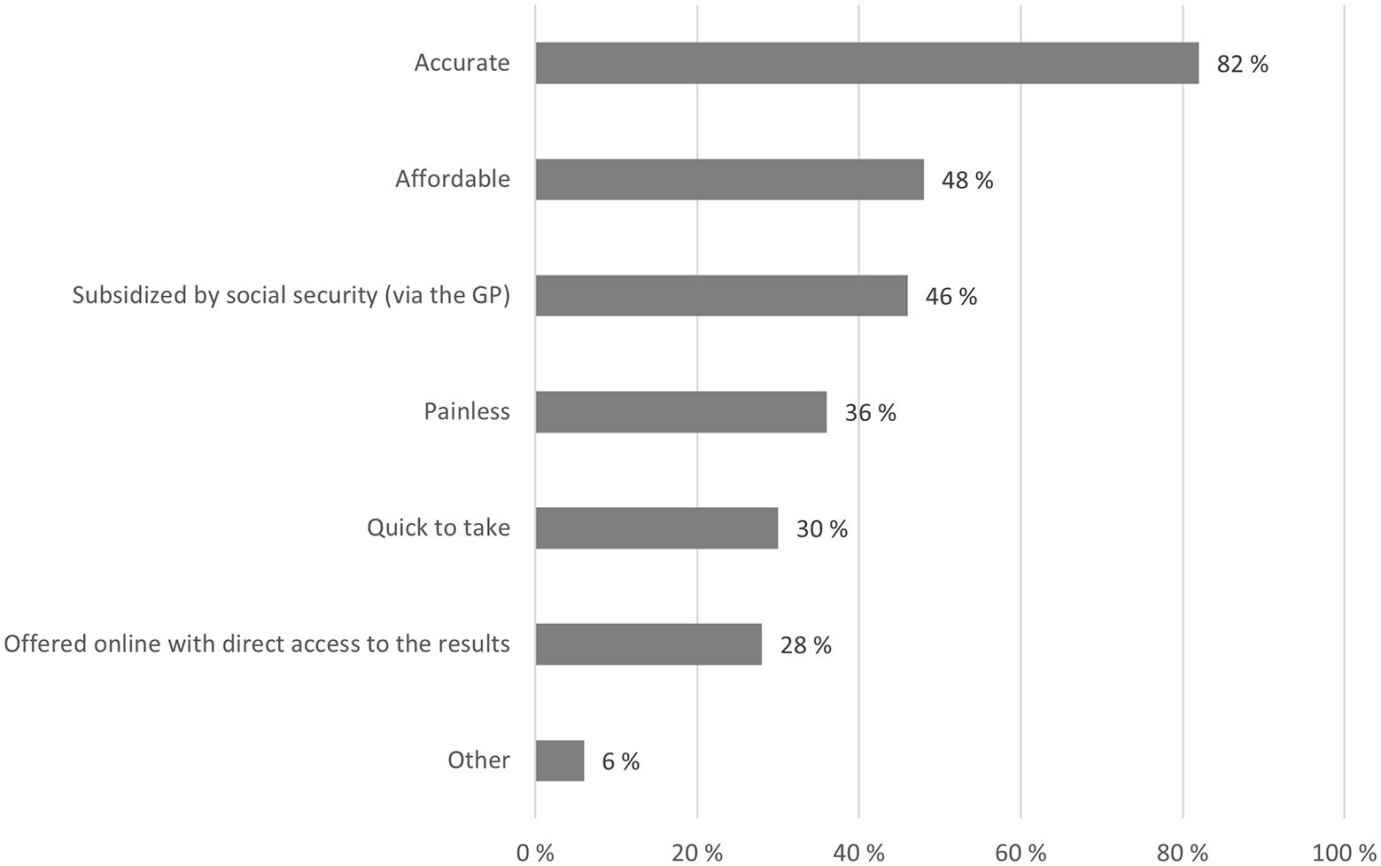
Brain health test criteria. Respondents could select up to three most important criteria for brain health tests. Numbers indicate the percentage of respondents selecting the given response categories.

Asked to indicate up to three of the most important criteria of a brain health test, respondents thought a test should be accurate (82%), affordable (48%), and be subsidized by social security (46%, see Figure 5).

Table 5 shows that young respondents thought it was more important that tests were affordable (OR 1.39, 99% CI 1.27–1.52), subsidized (OR 1.23, 99% CI 1.13–1.35) and not painful (OR 1.69, 99% CI 1.55–1.86) compared to older respondents. They were also less likely to think that tests were accurate (OR 0.63, 99% CI 0.56–0.71), available online (OR 0.52, 99% CI 0.47–0.58) or quick (OR 0.82, 99% CI 0.74–0.90) compared to older respondents.

**Table 5:** Test criteria.

**Variable**	**Characteristics**	**Accurate**			**Affordable**			**Subsidized**		
		**%**	**OR**	**99% CI**	**%**	**OR**	**99%CI**	**%**	**OR**	**99%CI**
Gender	Women	83.6			48.0			47.6		
	Men	81.2	0.84	0.77–0.92	51.2	1.14	1.06–1.22	43.4	0.84	0.79–0.90
	Other/Undisclosed	76.3	0.63	0.37–1.07	55.7	1.36	0.86–2.15	46.6	0.96	0.61–1.51
Age	>60 years	84.8			48.1			44.4		
	41–60 years	82.7	0.85	0.78–0.94	46.9	0.95	0.89–1.02	47.5	1.13	1.06–1.21
	≤ 40 years	77.9	0.63	0.56–0.71	56.2	1.39	1.27–1.52	49.6	1.23	1.13–1.35
Education	Higher education	83.4			49.2			44.7		
	Lower education	81.8	0.89	0.82–0.98	48.5	0.97	0.91–1.04	50.2	1.25	1.17–1.33
Cognitive health	Average or above	83.0			49.0			46.1		
	Below average	81.1	0.88	0.74–1.04	49.4	1.02	0.89–1.16	52.1	1.27	1.12–1.45
Mental health	Average or above	83.1			48.7			45.8		
	Below average	81.4	0.89	0.79–1.00	51.0	1.10	1.00–1.20	50.6	1.21	1.10–1.33
Caregiver exp.	No	81.2			48.9			47.5		
	Yes	84.8	1.29	1.19–1.41	49.0	1.00	0.94–1.07	45.2	0.91	0.85–0.97
Illness experience	No	82.9			50.2			44.2		
	Yes	82.9	1.00	0.92–1.09	47.1	0.88	0.83–0.94	49.7	1.24	1.17–1.33
Brain research exp.	No	80.1			48.2			48.4		
	Yes	86.7	1.62	1.49–1.77	50.1	1.08	1.01–1.15	43.8	0.83	0.78–0.88
Health care exp.	No	82.2			49.4			45.8		
	Yes	84.0	1.13	1.04–1.24	48.3	0.96	0.90–1.02	47.5	1.07	1.00–1.14
**Variable**	**Characteristics**	**Painless**			**Quick to take**			**Offered online**		
		**%**	**OR**	**99% CI**	**%**	**OR**	**99% CI**	**%**	**OR**	**99%CI**
Gender	Women	35.6			28.8			27.5		
	Men	33.9	0.93	0.86–1.00	33.0	1.22	1.13–1.31	28.9	1.07	0.99–1.16
	Other/Undisclosed	41.2	1.27	0.80–2.01	20.6	0.64	0.37–1.12	21.4	0.72	0.41–1.25
Age	>60 years	31.4			30.3			32.0		
	41–60 years	36.1	1.23	1.15–1.32	31.2	1.04	0.97–1.12	26.2	0.76	0.70–0.81
	≤ 40 years	43.7	1.69	1.55–1.86	26.3	0.82	0.74–0.90	19.7	0.52	0.47–0.58
Education	Higher education	35.6			29.6			27.6		
	Lower education	34.1	0.94	0.87–1.00	30.8	1.06	0.98–1.14	28.3	1.04	0.96–1.12
Cognitive health	Average or above	35.5			30.3			27.6		
	Below average	30.0	0.78	0.67–0.90	25.7	0.80	0.69–0.93	31.5	1.21	1.05–1.39
Mental health	Average or above	35.1			30.6			28.3		
	Below average	35.4	1.01	0.92–1.11	26.3	0.81	0.73–0.90	25.1	0.85	0.76–0.94
Caregiver exp.	No	36.9			30.0			27.4		
	Yes	33.1	0.85	0.79–0.90	29.9	1.00	0.93–1.07	28.3	1.05	0.98–1.12
Illness experience	No	36.1			30.5			27.7		
	Yes	33.8	0.90	0.85–0.97	29.3	0.94	0.88–1.01	28.0	1.02	0.95–1.09
Brain research exp.	No	35.9			31.2			27.0		
	Yes	34.2	0.93	0.87–0.99	28.4	0.87	0.82–0.94	28.9	1.10	1.02–1.17
Health care exp.	No	36.3			29.8			28.4		
	Yes	33.4	0.88	0.82–0.94	30.3	1.02	0.95–1.09	26.9	0.93	0.87–1.00
**Variable**	**Characteristics**	**Other**								
		**%**	**OR**	**99% CI**	**%**	**OR**	**99% CI**	**%**	**OR**	**99%CI**
Gender	Women	5.3								
	Men	5.1	0.97	0.83–1.14						
	Other/Undisclosed	6.9	1.32	0.54–3.24						
Age	>60 years	5.1								
	41–60 years	5.5	1.08	0.93–1.26						
	≤ 40 years	5.1	1.00	0.81–1.22						
Education	Higher education	6.1								
	Lower education	3.5	0.56	0.47–0.67						
Cognitive health	Average or above	5.2								
	Below average	5.5	1.05	0.79–1.40						
Mental health	Average or above	5.1								
	Below average	6.4	1.27	1.05–1.54						
Caregiver exp.	No	4.5								
	Yes	6.1	1.40	1.22–1.61						
Illness experience	No	4.7								
	Yes	6.1	1.31	1.14–1.51						
Brain research exp.	No	4.8								
	Yes	5.8	1.22	1.06–1.40						
Health care exp.	No	5.0								
	Yes	5.7	1.15	1.00–1.33						

% Indicates the proportion of participants selecting each characteristic. OR, Odds ratio; CI, Confidence interval.

Men thought it was more important that tests were quick (OR 1.22, 99% CI 1.13–1.31) than women. For respondents with lower education, subsidization of tests was more important (OR 1.25, 99% CI 1.17–1.33) than to those with higher education.

Respondents with poor cognitive health thought test subsidization was more important (OR 1.27, 99% CI 1.12–1.45) than those with good cognitive health, as did respondents with experience of long-standing illness or disability (OR 1.24, 99% CI 1.17–1.33) and respondents with poor mental health OR 1.21, 99% CI 1.10–1.33). Respondents with poor cognitive health thought it was less important that a test should be painless (OR 0.78, 99% CI 0.67–0.90) or quick (OR 0.80, 99% CI 0.69–0.93). Similarly, respondents with poor mental health thought it was less important that tests were quick (OR 0.81, 99% CI 0.73–0.90) than respondents with good mental health.

Test accuracy was more important to respondents who had cared for family members with brain disease (OR 1.29, 99% CI 1.19–1.41), and to respondents who had participated in brain research (OR 1.62, 99% CI 1.49–1.77), than other respondents.

## Discussion

### Summary of findings

This study is based on an international survey on public perspectives of brain health with 27,590 respondents and is one of the largest surveys to date. We explored public willingness to test for brain disease, and the motivation for doing so. Our main findings were that we found high public interest in brain health testing, even for diseases that cannot be treated or prevented. Further, those most interested in brain health testing were older (above 60), male, lower educated and with poorer cognitive health. The main reason for taking a test was to be able to act if they were found to be at risk of brain disease, such as changing lifestyle, seeking counseling or starting treatment. Most people said they would seek professional help and change their lifestyle if a test revealed they were at risk of brain disease. Of all the criteria for a good brain health test (price, invasiveness etc.), accuracy was rated as most important.

### Interest in brain health testing

In our study 91% of respondents stated they would definitely or probably take a brain health test to learn about the risk of developing a brain disease, and 86% would do so even if the disease was untreatable or unpreventable. These findings are consistent with previous studies that also have found high public interest in testing for brain diseases (17, 19, 20, 27, 34–39). The relatively high interest in testing observed here is likely due to this survey's focus on brain diseases at large, rather than on a specific brain disease. For instance, willingness to test for specific diseases such as Alzheimer's disease has been found to be somewhat less than for other diseases (34). We also found that our respondents were somewhat less willing to test for unpreventable or untreatable brain diseases, as identified in other studies (13, 35, 40), but interest is still high. Nevertheless, experience suggests that actual uptake of testing may be lower than initial interest, particularly in the absence of treatment options (39, 40). Caution is therefore needed in translating test willingness into actual testing behavior.

Our results indicate differences across demographic characteristics in the willingness to test for brain disease. We found that older people, men, lower educated, those with poorer cognitive health and those with first-hand experience with disease, either through personal health issues or through family members with brain disease, are most interested in testing for brain disease, including for untreatable or unpreventable brain diseases. Our finding that testing interest corresponds with pre-existing health issues or caregiving experience of family members with brain disease supports the notion that personal experience with brain disease and perceptions of personal risk increases the relative willingness to undergo testing for brain disease (8, 11). For example, previous studies find that interest in predictive testing for brain disease is high among people with a family history of dementia, and among people participating in brain research (13, 27, 41). Several studies also find that personal worry of developing brain disease increases test willingness (13, 20, 39, 40, 42).

On the other hand, we found that respondents with education and/or employment in healthcare were less interested in testing their risk of brain disease than those without. We can only speculate that health care professionals are less interested in testing because they are more aware of the limitations of current predictive tests. Relatedly, studies find that knowledge of medical testing, such as understanding the inherent prognostic uncertainty and limited clinical validity of predictive tests, reduces the motivation for brain-related testing (27, 39, 41), such that more knowledge can sometimes make people more skeptical. Previous studies are nevertheless highly inconsistent on the relative effect of various sociodemographic factors on testing interest (17, 19, 20, 35). Due to the non-representativeness of our survey sample, the sociodemographic variations in brain health test interest found here should be explored further in future studies.

### Motivations for brain health testing

An important contribution of this study was not only to investigate people's willingness to test their brain health, but to give insight into *why* people would be willing to do so. We found that the most important motivation for testing for brain disease was to respond if they were found to be at risk, for example through lifestyle changes. This finding concurs with studies that have shown how people are most inclined to change behavior if they are personally afflicted by brain disease or cognitive decline (8). However, while a few studies find that people alter their behavior after receiving brain health related test results, such as supplements intake and other lifestyle changes (43), most studies fail to identify significant behavioral changes (26, 44), commonly known as the intention-behavior gap (45).

Another central motivation for testing among our respondents was gaining information about personal brain health, either of personal risk of developing brain disease or of personal cognitive and mental condition, even for diseases that cannot be treated or prevented. Other studies concur on the central importance for consumers of obtaining personal information, even non-medical information, from brain-related tests (37, 42). Similarly, studies find that knowledge about personal risk of disease is valued for planning future care, healthcare decisions and late-life decisions (39, 41). Studies have also found that anticipation of last stages in life, and accessing healthcare, can be central motivations for undertaking testing for brain diseases (17, 20).

For the very few respondents in our study who were certain they did not want to take a brain health test, the primary motivation was not wanting to know about untreatable and/or unpreventable diseases, and not wanting to worry about something that might not happen. This supports previous research that dementia-worry can prevent some people from taking brain health tests all together (13, 20). On the whole however, our findings strengthen the notion that lay empowerment through information can be an important outcome of tests for brain disease risk.

### Premises for brain health testing

Of all the presented criteria, the most important characteristic of a brain health test was accuracy of results. Similarly, other studies have found that the willingness to take a brain health test is partly dependent on the validity of test results (27, 39, 41). Other central criteria were economic accessibility, either through affordable prices or subsidies. In our study cost-related criteria were more important to young respondents (40 years and below) and to respondents with low education levels and poor self-rated health.

### Study limitations

This study has several limitations. First, the survey departed from a real-life scenario by asking respondents to imagine an unspecified and hypothetical test for brain disease that is currently unavailable. The respondents were not given the type of information that those who undergo real-life predictive testing in genetics must have before they undergo tests, such as for early detection of Alzheimer's for example. Other studies have shown that the willingness to undertake hypothetical tests is greater than actual test willingness when such tests are developed (39). Secondly, the respondents in this survey are unrepresentative of the general population because many were conveniently recruited via brain research organizations, institutes and research networks connected to the Lifebrain project. In line with most other relevant studies, respondents are more likely to be female, above middle age and have higher education. Moreover, in this study close to six of 10 had been recruited via brain research registries, close to half had experience with taking care of family members with brain disease, and about four out of 10 respondents had an education or employment in healthcare or had participated in brain research. Most of the respondents were in Europe, largely due to the sampling procedure and the European-based survey stakeholders (12, 31). These sociodemographic and individual characteristics may thus be due to self-selection bias and not give a true reflection of the general public's perceptions of brain health at large.

To further probe the effect of self-selection, we compared participants recruited through research registers and those who responded independently from the same countries. As expected, a larger proportion of the registry participants had taken part in brain research, and the more ostensibly respondents were engaged with brain health, the more they were likely to want to take a hypothetical brain health test. Even the “non-organised” participants who were not part of a research registry, had a large proportion of brain research participants (>25%)—more than one would expect in the general population. Both groups—those recruited through registers and the “non-organised”—were mostly highly educated, middle-aged, or older, and female, and therefore not representative of the general population. Despite this selectivity, the respondents were also people who had access to the internet and an interest in the topic, as well as the motivation to spend time in answering questions anonymously, without any further financial or other external motivation. The respondents were sufficiently interested in completing the questionnaire on their own initiative, and they therefore represent the stratum of society that is more informed about brain health and also likely politically more engaged than the average citizen.

## Conclusions and implications for future policy and research

Despite a self-selective sample with e.g., a large proportion of higher educated female respondents already interested in brain health, we believe this study provides several key insights that may be relevant for health authorities and policy makers, as interested citizens are likely to influence public opinion disproportionately. Firstly, we find that the vast majority of respondents wanted to take a simple hypothetical test to detect risk of brain disease. Test interest was high among respondents with personal experience of illness or of taking care of next of kin with brain disease. Given the expected increase in people with personal or family-related experience in brain diseases (3) our results indicate significant public demand for tests for brain diseases in the years to come.

### Demand for personal information

Importantly, our results also show that for many respondents, obtaining personal brain health information was by itself a goal, regardless of preventative opportunities. While expressed interest in testing does not necessarily translate into actual testing behavior, this might change as less invasive and more accessible tests are becoming available. Studies have shown that the availability of high-quality information on the limitations of medical tests can adjust public expectations and thereby reduce the willingness to undergo testing. Relatedly, we find that respondents with experience and/or education in health care were less interested in brain health testing than other respondents. Studies also indicate the importance of access to high quality information after receiving test results to limit adverse reactions. A key task for both providers of medical tests and health authorities is hence to increase public education on the limitations and implications of medical testing and thoughtful communication of test results.

### Motivations for behavioral change

Secondly, we find that most of the respondents indicate that a key motivational factor for testing for brain disease is changing their lifestyle. While research shows that intention to change behavior increases the likelihood of doing so, people generally do not change their behavior after receiving test results, despite their intentions to do so (21). Test results can also potentially reduce motivation to change behavior (21) or inspire changes that are undocumented and not necessarily beneficial (40, 43). Nevertheless, thoughtful communication of test results, combined with personalized recommendations of evidence-based interventions, can increase the likelihood of behavioral change (21). A qualitative study for instance suggests that access to high-quality information on recommended activities to strengthen brain health can narrow the intention-behavior gap by facilitating long-term behavioral changes (42). Similarly, an Australian study found that both intentions and actions to improve brain health were associated with knowledge about dementia risk reduction (15). A pilot study of the GBHS found that personalized advice should also incorporate individual differences of motivations, triggers and capacities for lifestyle changes (13). Consequently, a second central task for health authorities should be to provide easily accessible public information on recommended behavioral changes to optimize brain health and reduce risk of developing brain disease.

### Increased pressures on public health systems

Other central motivational factors for undertaking testing were seeking professional help and treatment if found to be necessary. This finding suggests that future public demand for professional follow up could be significant should tests for brain disease risk become widely available to the general public, in particular since risk factors are also present in people who never develop brain disease (6, 25). Widespread public brain health testing could hence both overwhelm the health care system and result in unnecessary and potentially harmful overtreatment. Given that commercial tests are often provided outside a framework of personal feedback and follow-up, the provision of high-quality information on medical testing and lifestyle changes could also help mitigate such unwanted consequences.

### Future directions

In sum, considering the increasing amount of consumer tests entering the market, paired with optimistic, popularized representations of medical testing by the media, there is a need to foster realistic public expectations of brain health testing. Our study suggests a critical role of public authorities to educate the public on brain health testing and the provision of public brain health interventions and activities. Recognizing the difficulty of succeeding with lifestyle changes, we furthermore call for more research into factors that facilitate behavioral change for looking after ones' brain health. At the same time, public perspectives of brain health are evolving, and future research should therefore repeat this kind of survey at regular intervals to chart changes in perceptions, also in other parts of the world and in different segments of the population.

## Data availability statement

The data is publicly available with a CC-BY licence through GItHub as a tab-separated file or as an R data package, https://github.com/Lifebrain/gbhs. All source materials for data retrieval, cleanup, modelling and visualisation is also contained in the same repository. All these materials can also be found with their doi: 10.5281/zenodo.7116985.

## Ethics statement

The studies involving human participants were reviewed and approved by Regional Committees for Medical and Health Research Ethics in Norway (2017/653 REK SørØst B). The survey was also approved by the University of Oxford Medical Sciences Interdivisional Research Ethics Committee (R67364/RE001) and the Medical Ethics Review Committee of VU University Medical Centre in the Netherlands for permission to disseminate the survey. The patients/participants provided their written informed consent to participate in this study.

## Author contributions

RC and NF drafted and finalized the manuscript. IB-L led the study. IB-L, AM, BF, SS, CS-P, CD, WB, EZ, DB-F, PG, LN, KM, SD, and KW conceived the study and developed the survey. RC, IB-L, AM, BF, KE, EZ, CD, WB, DB-F, CS-P, and KW were involved in data collection and analysis. AM, EZ, and KE helped with the core statistical analysis. RC, NF, IB-L, KE, BF, TR, CD, SS, WB, CS-P, DB-F, PG, AF, UL, KM, and KW substantively revised the manuscript prior to submission. All authors contributed to the article and approved the submitted version.

## Funding

This research was funded by the EU Horizon 2020 Grant: Healthy minds 0–100 years: Optimising the use of European brain imaging cohorts (Lifebrain), Grant Agreement No. 732592.

## Conflict of interest

The authors declare that the research was conducted in the absence of any commercial or financial relationships that could be construed as a potential conflict of interest.

## Publisher's note

All claims expressed in this article are solely those of the authors and do not necessarily represent those of their affiliated organizations, or those of the publisher, the editors and the reviewers. Any product that may be evaluated in this article, or claim that may be made by its manufacturer, is not guaranteed or endorsed by the publisher.

## Supplementary material

Supplementary Material 1 Binary and continuous outcome models, and descriptive outcomes of sub-questions with Odds Ratios and Confidence Intervals.
