# Supplementary material for: People's interest in brain health testing: Findings from an international, online cross-sectional survey
Source: Front Public Health. 2022 Oct 20;10:998302. doi: 10.3389/fpubh.2022.998302 (PMC9631023; doi:10.3389/fpubh.2022.998302)
Supplement: Supplementary Material 1 — Binary and continuous outcome models, and descriptive outcomes of sub-questions with Odds Ratios and Confidence Intervals. [file Data_Sheet_1.pdf]

Carver et al. 2022. People's interest in brain health testing: Findings from an international, online cross-sectional survey. Frontiers in Public Health. DOI 10.3389/fpubh.2022.998302

LIFEBRAIN GLOBAL BRAIN HEALTH SURVEY

TABLE OF CONTENTS:

Table of contents: 1

SELECTED COMPARISONS OF BINARY VS. CONTINUOUS OUTCOME MODELS. 3

Table 1: Question 1 (Imagine a simple brain health test to learn about risk of developing a brain disease, would you wish to take such a test?): Comparison of binary vs. continuous outcome models. 3

Table 2: Question 2 (Would you take a test even if it provides information about a disease that cannot be prevented or treated?): Comparison of binary vs. continuous outcome models. 4

Table 3: Question 5 (Imagine you undergo a brain health test, and it shows that you have a risk of developing brain disease): Comparison of binary vs. continuous outcome models. 5

DESCRIPTIVE OUTCOME OF SUB-QUESTIONS WITH ODDS RATIOS AND CONFIDENCE INTERVALS 6

Table 4: Question 1 Imagine a simple brain health test to learn about risk of developing a brain disease. Would you wish to take such a test? 6

Table 5: Question 2 (Would you take a test even if it provides information about a disease that cannot be prevented or treated?) 7

Table 6: Question 3a (Why would you take a brain health test? - To get information about my cognitive and mental health) 8

Table 7: Question 3b (Why would you take a brain health test? - To determine my risk of developing a brain disease) 9

Table 8: Question 3c (Why would you take a brain health test? - To respond if I am at risk, e.g. change my lifestyle, seek counselling, or start treatment) 10

Table 9: Question 3d (Why would you take a brain health test? - To prepare myself for the future (e.g. inform my family about the risk) 11

Table 10: Question 3e (Why would you take a brain health test? – For another reason) 12

Table 11: Question 4a (Why would you not take a brain health test? - I do not want to worry about something that may not happen) 13

Table 12: Question 4b (Why would you not take a brain health test? - I do not want to know about a disease that could not be prevented or treated) 14

Table 13 Question 4c (Why would you not take a brain health test? - I would be frightened by the result)......... 15

Table 14: Question 4d (Why would you not take a brain health test? - There is nothing I can do for my brain health anyway) 16

Table 15: Question 4e (Why would you not take a brain health test? – For other reasons) 17

Table 16: Question 5a (Imagine you undergo a brain health test, and it shows that you have a risk of developing brain disease. What would be your most likely reaction? - I would seek professional help (e.g., my doctor)) 18

Table 17: Question 5b (Imagine you undergo a brain health test, and it shows that you have a risk of developing brain disease. What would be your most likely reaction? - I would seek advice from family and friends) 19

Table 18: Question 5c (Imagine you undergo a brain health test, and it shows that you have a risk of developing brain disease. What would be your most likely reaction? - I would seek information online/at the library)........ 20

Table 19: Question 5d (Imagine you undergo a brain health test, and it shows that you have a risk of developing brain disease. What would be your most likely reaction? - I would change my lifestyle if required).................... 21

Table 20: Question 5e (Imagine you undergo a brain health test, and it shows that you have a risk of developing brain disease. What would be your most likely reaction? - I would plan for the future) 22

Table 21: Question 6a (Brain Health Test should be affordable) 23

Table 22: Question 6b (Brain Health Test should be quick to take) 24

Table 23: Question 6c (Brain Health Test should be accurate) 25

Table 24: Question 6d (Brain Health Test should be painless) 26

Table 25: Question 6e (Brain Health Test should be subsidized by social security) 27

Table 26: Question 6f (Brain Health Test should be offered online) 28

Table 27: Question 6g (Brain Health Test should have [other] characteristic) 29

Selected Comparisons of binary vs. continuous outcome models.

Table 1: Question 1 (Imagine a simple brain health test to learn about risk of developing a brain disease, would you wish to take such a test?): Comparison of binary vs. continuous outcome models.

			Binary models				Continuous models			
Key	factor	term	log odds	std.error	statistic	p.value	beta	std.error	statistic	p.value
Probability of taking a brain health test	mental health	Below average	-0.001	0.062	-0.01	0.992	0.025	0.012	2.042	0.041

[a] Models and groups where the sign of the beta (estimate) between continuous and binary outcome measures are in opposite directions.

TABLE 2: QUESTION 2 (WOULD YOU TAKE A TEST EVEN IF IT PROVIDES INFORMATION ABOUT A DISEASE THAT CANNOT BE PREVENTED OR TREATED?): COMPARISON OF BINARY VS. CONTINUOUS OUTCOME MODELS.

			Binary models				Continuous models			
Key	factor	term	log odds	std.error	statistic	p.value	beta	std.error	statistic	p.value
Probability of taking a brain health test even if no treatment is available	Relationship	Stable	0.027	0.037	0.745	0.456	-0.009	0.009	-1.033	0.302

[a] Models and groups where the sign of the beta (estimate) between continuous and binary outcome measures are in opposite directions.

Table 3: Question 5 (Imagine you undergo a brain health test, and it shows that you have a risk of developing brain disease): Comparison of binary vs. continuous outcome models.

			Binary models				Continuous models			
Key	factor	term	log odds	std.error	statistic	p.value	beta	std.error	statistic	p.value
Plan for the future	Age	41-60	0.077	0.064	1.201	0.230	-0.023	0.008	-2.875	0.004
Seek information online/ at the library	Gender	Other/ Undisclosed	-0.098	0.317	-0.311	0.756	0.015	0.065	0.227	0.820
	Illness experience	Yes	-0.001	0.043	-0.018	0.986	0.016	0.009	1.726	0.084
Seek professional help	Brain disease caregiver	Yes	-0.070	0.070	-1.002	0.317	0.003	0.006	0.512	0.609
	Cognitive health	Below average	-0.057	0.144	-0.395	0.693	0.000	0.013	0.015	0.988
Seek advice from family and friends	Relationship	Stable	-0.016	0.026	-0.594	0.553	0.002	0.011	0.173	0.863

[a] Models and groups where the sign of the beta (estimate) between continuous and binary outcome measures are in opposite directions.

Descriptive outcome of Sub-Questions with Odds Ratios and Confidence Intervals

Table 4: Question 1 Imagine a simple brain health test to learn about risk of developing a brain disease. Would you wish to take such a test?

Group	Positive Response	n	OR	Lower CI (99%)	Upper CI (99%)
**Age**					
>= 61	11 812 (92.6%)	12 760			
<= 40	4 007 (89.0%)	4 502	0.65	0.56	0.76
41-60	9 311 (90.2%)	10 328	0.73	0.65	0.83
**Brain Disease Caregiver**					
No	13 399 (90.8%)	14 762			
Yes	11 731 (91.4%)	12 828	1.09	0.97	1.21
**Brain Research Participant**					
No	14 129 (90.2%)	15 671			
Yes	11 001 (92.3%)	11 919	1.31	1.17	1.46
**Cognitive Health**					
Average or above	23 574 (90.9%)	25 929			
Below average	1 556 (93.7%)	1 661	1.48	1.13	1.93
**Education**					
Higher	17 050 (90.1%)	18 925			
Lower	8 080 (93.2%)	8 665	1.52	1.34	1.72
**Gender**					
Woman	17 660 (90.0%)	19 626			
Other/Undisclosed	106 (80.9%)	131	0.47	0.27	0.84
Man	7 364 (94.0%)	7 833	1.75	1.52	2.01
**Healthcare Experience**					
No	15 636 (92.2%)	16 955			
Yes	9 494 (89.3%)	10 635	0.70	0.63	0.78
**Illness Experience**					
No	14 868 (90.4%)	16 451			
Yes	10 262 (92.1%)	11 139	1.25	1.11	1.40
**Mental Health**					
Average or above	21 822 (91.1%)	23 958			
Below average	3 308 (91.1%)	3 632	1.00	0.85	1.17
**Relationship**					
Not stable	11 085 (91.0%)	12 177			
Stable	14 045 (91.1%)	15 413	1.01	0.91	1.13

TABLE 5: QUESTION 2 (WOULD YOU TAKE A TEST EVEN IF IT PROVIDES INFORMATION ABOUT A DISEASE THAT CANNOT BE PREVENTED OR TREATED?)

Group	Positive Response	n	OR	Lower CI (99%)	Upper CI (99%)
**Age**					
>= 61	10 446 (88.4%)	11 812			
<= 40	3 281 (81.9%)	4 007	0.59	0.52	0.67
41-60	7 953 (85.4%)	9 311	0.77	0.69	0.85
**Brain Disease Caregiver**					
No	11 423 (85.3%)	13 399			
Yes	10 257 (87.4%)	11 731	1.20	1.09	1.32
**Brain Research Participant**					
No	11 951 (84.6%)	14 129			
Yes	9 729 (88.4%)	11 001	1.39	1.26	1.54
**Cognitive Health**					
Average or above	20 251 (85.9%)	23 574			
Below average	1 429 (91.8%)	1 556	1.85	1.45	2.36
**Education**					
Higher	14 504 (85.1%)	17 050			
Lower	7 176 (88.8%)	8 080	1.39	1.25	1.55
**Gender**					
Woman	14 922 (84.5%)	17 660			
Other/Undisclosed	91 (85.8%)	106	1.11	0.54	2.29
Man	6 667 (90.5%)	7 364	1.76	1.56	1.97
**Healthcare Experience**					
No	13 740 (87.9%)	15 636			
Yes	7 940 (83.6%)	9 494	0.71	0.64	0.78
**Illness Experience**					
No	12 595 (84.7%)	14 868			
Yes	9 085 (88.5%)	10 262	1.39	1.26	1.54
**Mental Health**					
Average or above	18 791 (86.1%)	21 822			
Below average	2 889 (87.3%)	3 308	1.11	0.96	1.28
**Relationship**					
Not stable	9 543 (86.1%)	11 085			
Stable	12 137 (86.4%)	14 045	1.03	0.93	1.13

Table 6: Question 3a (Why would you take a brain health test? - To get information about my cognitive and mental health)

Group	Positive Response	n	OR	Lower CI (99%)	Upper CI (99%)
**Age**					
>= 61	3 975 (31.2%)	12 760			
<= 40	1 878 (41.7%)	4 502	1.74	1.58	1.91
41-60	3 052 (29.6%)	10 328	0.96	0.89	1.04
**Brain Disease Caregiver**					
No	5 222 (35.4%)	14 762			
Yes	3 683 (28.7%)	12 828	0.72	0.67	0.77
**Brain Research Participant**					
No	5 239 (33.4%)	15 671			
Yes	3 666 (30.8%)	11 919	0.85	0.79	0.91
**Cognitive Health**					
Average or above	8 333 (32.1%)	25 929			
Below average	572 (34.4%)	1 661	1.06	0.92	1.22
**Education**					
Higher	6 146 (32.5%)	18 925			
Lower	2 759 (31.8%)	8 665	0.92	0.85	0.99
**Gender**					
Woman	6 077 (31.0%)	19 626			
Other/Undisclosed	48 (36.6%)	131	1.58	0.95	2.61
Man	2 780 (35.5%)	7 833	1.16	1.07	1.25
**Healthcare Experience**					
No	5 543 (32.7%)	16 955			
Yes	3 362 (31.6%)	10 635	1.00	0.93	1.07
**Illness Experience**					
No	5 190 (31.5%)	16 451			
Yes	3 715 (33.4%)	11 139	1.06	0.99	1.13
**Mental Health**					
Average or above	7 585 (31.7%)	23 958			
Below average	1 320 (36.3%)	3 632	1.25	1.13	1.38
**Relationship**					
Not stable	4 288 (35.2%)	12 177			
Stable	4 617 (30.0%)	15 413	0.78	0.72	0.83

Table 7: Question 3b (Why would you take a brain health test? - To determine my risk of developing a brain disease)

Group	Positive Response	n	OR	Lower CI (99%)	Upper CI (99%)
**Age**					
>= 61	4 570 (35.8%)	12 760			
<= 40	1 291 (28.7%)	4 502	0.75	0.68	0.83
41-60	3 514 (34.0%)	10 328	0.96	0.89	1.03
**Brain Disease Caregiver**					
No	4 799 (32.5%)	14 762			
Yes	4 576 (35.7%)	12 828	1.15	1.07	1.23
**Brain Research Participant**					
No	5 216 (33.3%)	15 671			
Yes	4 159 (34.9%)	11 919	1.04	0.97	1.11
**Cognitive Health**					
Average or above	8 794 (33.9%)	25 929			
Below average	581 (35.0%)	1 661	1.00	0.87	1.15
**Education**					
Higher	6 178 (32.6%)	18 925			
Lower	3 197 (36.9%)	8 665	1.15	1.07	1.24
**Gender**					
Woman	6 624 (33.8%)	19 626			
Other/Undisclosed	27 (20.6%)	131	0.57	0.32	1.01
Man	2 724 (34.8%)	7 833	0.98	0.91	1.05
**Healthcare Experience**					
No	5 852 (34.5%)	16 955			
Yes	3 523 (33.1%)	10 635	0.99	0.92	1.06
**Illness Experience**					
No	5 621 (34.2%)	16 451			
Yes	3 754 (33.7%)	11 139	0.95	0.89	1.02
**Mental Health**					
Average or above	8 236 (34.4%)	23 958			
Below average	1 139 (31.4%)	3 632	0.87	0.78	0.96
**Relationship**					
Not stable	3 961 (32.5%)	12 177			
Stable	5 414 (35.1%)	15 413	1.13	1.05	1.21

Table 8: Question 3c (Why would you take a brain health test? - To respond if I am at risk, e.g. change my lifestyle, seek counselling, or start treatment)

Group	Positive Response	n	OR	Lower CI (99%)	Upper CI (99%)
**Age**					
>= 61	5 897 (46.2%)	12 760			
<= 40	2 404 (53.4%)	4 502	1.50	1.37	1.66
41-60	4 996 (48.4%)	10 328	1.16	1.08	1.25
**Brain Disease Caregiver**					
No	7 353 (49.8%)	14 762			
Yes	5 944 (46.3%)	12 828	0.84	0.79	0.90
**Brain Research Participant**					
No	7 561 (48.2%)	15 671			
Yes	5 736 (48.1%)	11 919	0.95	0.89	1.01
**Cognitive Health**					
Average or above	12 566 (48.5%)	25 929			
Below average	731 (44.0%)	1 661	0.78	0.68	0.89
**Education**					
Higher	9 372 (49.5%)	18 925			
Lower	3 925 (45.3%)	8 665	0.77	0.72	0.83
**Gender**					
Woman	9 373 (47.8%)	19 626			
Other/Undisclosed	58 (44.3%)	131	1.07	0.64	1.77
Man	3 866 (49.4%)	7 833	0.98	0.91	1.05
**Healthcare Experience**					
No	8 210 (48.4%)	16 955			
Yes	5 087 (47.8%)	10 635	1.04	0.98	1.12
**Illness Experience**					
No	7 981 (48.5%)	16 451			
Yes	5 316 (47.7%)	11 139	0.93	0.87	0.99
**Mental Health**					
Average or above	11 539 (48.2%)	23 958			
Below average	1 758 (48.4%)	3 632	1.01	0.92	1.11
**Relationship**					
Not stable	6 003 (49.3%)	12 177			
Stable	7 294 (47.3%)	15 413	0.91	0.86	0.98

Table 9: Question 3d (Why would you take a brain health test? - To prepare myself for the future (e.g. inform my family about the risk)

**Group**	**Positive Response**	**n**	**OR**	**Lower CI (99%)**	**Upper CI (99%)**
**Age**					
>= 61	2 775 (21.7%)	12 760			
<= 40	542 (12.0%)	4 502	0.51	0.45	0.58
41-60	1 766 (17.1%)	10 328	0.76	0.70	0.83
**Brain Disease Caregiver**					
No	2 328 (15.8%)	14 762			
Yes	2 755 (21.5%)	12 828	1.46	1.35	1.58
**Brain Research Participant**					
No	2 432 (15.5%)	15 671			
Yes	2 651 (22.2%)	11 919	1.53	1.41	1.66
**Cognitive Health**					
Average or above	4 756 (18.3%)	25 929			
Below average	327 (19.7%)	1 661	1.05	0.89	1.24
**Education**					
Higher	3 514 (18.6%)	18 925			
Lower	1 569 (18.1%)	8 665	0.93	0.85	1.01
**Gender**					
Woman	3 620 (18.4%)	19 626			
Other/Undisclosed	30 (22.9%)	131	1.53	0.88	2.68
Man	1 433 (18.3%)	7 833	0.94	0.86	1.03
**Healthcare Experience**					
No	3 166 (18.7%)	16 955			
Yes	1 917 (18.0%)	10 635	1.00	0.92	1.08
**Illness Experience**					
No	2 919 (17.7%)	16 451			
Yes	2 164 (19.4%)	11 139	1.09	1.01	1.19
**Mental Health**					
Average or above	4 479 (18.7%)	23 958			
Below average	604 (16.6%)	3 632	0.86	0.76	0.98
**Relationship**					
Not stable	2 075 (17.0%)	12 177			
Stable	3 008 (19.5%)	15 413	1.18	1.09	1.28

TABLE 10: QUESTION 3E (WHY WOULD YOU TAKE A BRAIN HEALTH TEST? – FOR ANOTHER REASON)

Group	Positive Response	n	OR	Lower CI (99%)	Upper CI (99%)
**Age**					
>= 61	252 (2.0%)	12 760			
<= 40	65 (1.4%)	4 502	0.76	0.53	1.09
41-60	238 (2.3%)	10 328	1.20	0.95	1.52
**Brain Disease Caregiver**					
No	230 (1.6%)	14 762			
Yes	325 (2.5%)	12 828	1.63	1.30	2.04
**Brain Research Participant**					
No	297 (1.9%)	15 671			
Yes	258 (2.2%)	11 919	1.12	0.90	1.40
**Cognitive Health**					
Average or above	486 (1.9%)	25 929			
Below average	69 (4.2%)	1 661	2.20	1.57	3.09
**Education**					
Higher	333 (1.8%)	18 925			
Lower	222 (2.6%)	8 665	1.42	1.13	1.78
**Gender**					
Woman	397 (2.0%)	19 626			
Other/Undisclosed	154 (2.0%)	7 833	0.93	0.73	1.19
Man	4 (3.1%)	131	1.71	0.45	6.39
**Healthcare Experience**					
No	371 (2.2%)	16 955			
Yes	184 (1.7%)	10 635	0.81	0.64	1.03
**Illness Experience**					
No	256 (1.6%)	16 451			
Yes	299 (2.7%)	11 139	1.71	1.37	2.14
**Mental Health**					
Average or above	428 (1.8%)	23 958			
Below average	127 (3.5%)	3 632	2.00	1.53	2.60
**Relationship**					
Not stable	249 (2.0%)	12 177			
Stable	306 (2.0%)	15 413	0.97	0.78	1.21

TABLE 11: QUESTION 4A (WHY WOULD YOU NOT TAKE A BRAIN HEALTH TEST? - I DO NOT WANT TO WORRY ABOUT SOMETHING THAT MAY NOT HAPPEN)

Group	Positive Response	n	OR	Lower CI (99%)	Upper CI (99%)
**Age**					
>= 61	261 (27.5%)	261 (27.5%)			
<= 40	133 (26.9%)	133 (26.9%)	0.97	0.70	1.33
41-60	269 (26.5%)	269 (26.5%)	0.95	0.73	1.23
**Brain Disease Caregiver**					
No	381 (28.0%)	1 363			
Yes	282 (25.7%)	1 097	0.89	0.70	1.13
**Brain Research Participant**					
No	451 (29.2%)	1 542			
Yes	212 (23.1%)	918	0.73	0.57	0.93
**Cognitive Health**					
Average or above	638 (27.1%)	2 355			
Below average	25 (23.8%)	105	0.84	0.46	1.54
**Education**					
Higher	464 (24.7%)	1 875			
Lower	199 (34.0%)	585	1.57	1.20	2.04
**Gender**					
Woman	525 (26.7%)	1 966			
Other/Undisclosed	10 (40.0%)	25	1.83	0.63	5.29
Man	128 (27.3%)	469	1.03	0.76	1.39
**Healthcare Experience**					
No	368 (27.9%)	1 319			
Yes	295 (25.9%)	1 141	0.90	0.71	1.14
**Illness Experience**					
No	422 (26.7%)	1 583			
Yes	241 (27.5%)	877	1.04	0.82	1.33
**Mental Health**					
Average or above	580 (27.2%)	2 136			
Below average	83 (25.6%)	324	0.92	0.65	1.31
**Relationship**					
Not stable	277 (25.4%)	1 092			
Stable	386 (28.2%)	1 368	1.16	0.91	1.47

TABLE 12: QUESTION 4B (WHY WOULD YOU NOT TAKE A BRAIN HEALTH TEST? - I DO NOT WANT TO KNOW ABOUT A DISEASE THAT COULD NOT BE PREVENTED OR TREATED)

Group	Positive Response	n	OR	Lower CI (99%)	Upper CI (99%)
**Age**					
>= 61	570 (60.1%)	948			
<= 40	291 (58.8%)	495	0.95	0.71	1.27
41-60	572 (56.2%)	1017	0.85	0.67	1.08
**Brain Disease Caregiver**					
No	759 (55.7%)	1 363			
Yes	674 (61.4%)	1 097	1.27	1.02	1.57
**Brain Research Participant**					
No	889 (57.7%)	1 542			
Yes	544 (59.3%)	918	1.07	0.86	1.33
**Cognitive Health**					
Average or above	1 372 (58.3%)	2 355			
Below average	61 (58.1%)	105	0.99	0.59	1.67
**Education**					
Higher	1 144 (61.0%)	1 875			
Lower	289 (49.4%)	585	0.62	0.49	0.80
**Gender**					
Woman	1 167 (59.4%)	1 966			
Other/Undisclosed	8 (32.0%)	25	0.32	0.11	0.98
Man	258 (55.0%)	469	0.84	0.64	1.09
**Healthcare Experience**					
No	747 (56.6%)	1 319			
Yes	686 (60.1%)	1 141	1.15	0.93	1.43
**Illness Experience**					
No	931 (58.8%)	1 583			
Yes	502 (57.2%)	877	0.94	0.75	1.17
**Mental Health**					
Average or above	1 241 (58.1%)	2 136			
Below average	192 (59.3%)	324	1.05	0.77	1.43
**Relationship**					
Not stable	624 (57.1%)	1 092			
Stable	809 (59.1%)	1 368	1.09	0.88	1.34

Table 13 Question 4c (Why would you not take a brain health test? - I would be frightened by the result)

Group	Positive Response	n	OR	Lower CI (99%)	Upper CI (99%)
**Age**					
= 61	84 (8.9%)	948			
<= 40	76 (15.4%)	495	1.87	1.21	2.89
41-60	120 (11.8%)	1 017	1.38	0.93	2.03
**Brain Disease Caregiver**					
No	171 (12.5%)	1 363			
Yes	109 (9.9%)	1 097	0.77	0.55	1.08
**Brain Research Participant**					
No	198 (12.8%)	1 542			
Yes	82 (8.9%)	918	0.67	0.47	0.95
**Cognitive Health**					
Average or above	265 (11.3%)	2 355			
Below average	15 (14.3%)	105	1.31	0.63	2.75
**Education**					
Higher	194 (10.3%)	1 875			
Lower	86 (14.7%)	585	1.49	1.04	2.14
**Gender**					
Woman	231 (11.7%)	1 966			
Other/Undisclosed	4 (16.0%)	25	1.43	0.35	5.91
Man	45 (9.6%)	469	0.80	0.51	1.24
**Healthcare Experience**					
No	161 (12.2%)	1 319			
Yes	119 (10.4%)	1 141	0.84	0.60	1.17
**Illness Experience**					
No	172 (10.9%)	1 583			
Yes	108 (12.3%)	877	1.15	0.82	1.61
**Mental Health**					
Average or above	224 (10.5%)	2 136			
Below average	56 (17.3%)	324	1.78	1.17	2.72
**Relationship**					
Not stable	118 (10.8%)	1 092			
Stable	162 (11.8%)	1 368	1.11	0.80	1.54

TABLE 14: QUESTION 4D (WHY WOULD YOU NOT TAKE A BRAIN HEALTH TEST? - THERE IS NOTHING I CAN DO FOR MY BRAIN HEALTH ANYWAY)

Group	Positive Response	n	OR	Lower CI (99%)	Upper CI (99%)
**Age**					
>= 61	23 (2.4%)	948			
<= 40	2 (0.4%)	495	0.16	0.02	1.10
41-60	10 (1.0%)	1 017	0.40	0.15	1.07
**Brain Disease Caregiver**					
No	21 (1.5%)	1 363			
Yes	14 (1.3%)	1 097	0.83	0.34	2.02
**Brain Research Participant**					
No	26 (1.7%)	1 542			
Yes	9 (1.0%)	918	0.58	0.21	1.58
**Cognitive Health**					
Average or above	30 (1.3%)	2 355			
Below average	5 (4.8%)	105	3.87	1.08	13.85
**Education**					
Higher	18 (1.0%)	1 875			
Lower	17 (2.9%)	585	3.09	1.28	7.45
**Gender**					
Woman	19 (1.0%)	1 966			
Other/Undisclosed	1 (4.0%)	25	4.27	0.29	63.50
Man	15 (3.2%)	469	3.39	1.37	8.34
**Healthcare Experience**					
No	23 (1.7%)	1 319			
Yes	12 (1.1%)	1 141	0.60	0.24	1.51
**Illness Experience**					
No	24 (1.5%)	1 583			
Yes	11 (1.3%)	877	0.83	0.32	2.12
**Mental Health**					
Average or above	29 (1.4%)	2 136			
Below average	6 (1.9%)	324	1.37	0.43	4.41
**Relationship**					
Not stable	14 (1.3%)	1 092			
Stable	21 (1.5%)	1 368	1.20	0.49	2.94

TABLE 15: QUESTION 4E (WHY WOULD YOU NOT TAKE A BRAIN HEALTH TEST? – FOR OTHER REASONS)

Group	Positive Response	n	OR	Lower CI (99%)	Upper CI (99%)
**Age**					
>= 61	87 (9.2%)	948			
<= 40	44 (8.9%)	495	0.97	0.59	1.59
41-60	136 (13.4%)	1 017	1.53	1.05	2.22
**Brain Disease Caregiver**					
No	156 (11.4%)	1 363			
Yes	111 (10.1%)	1 097	0.87	0.62	1.22
**Brain Research Participant**					
No	136 (8.8%)	1 542			
Yes	131 (14.3%)	918	1.72	1.23	2.41
**Cognitive Health**					
Average or above	257 (10.9%)	2 355			
Below average	10 (9.5%)	105	0.86	0.36	2.06
**Education**					
Higher	200 (10.7%)	1 875			
Lower	67 (11.5%)	585	1.08	0.74	1.59
**Gender**					
Woman	201 (10.2%)	1 966			
Other/Undisclosed	5 (20%)	25	2.20	0.60	8.09
Man	61 (13.0%)	469	1.31	0.88	1.96
**Healthcare Experience**					
No	140 (10.6%)	1 319			
Yes	127 (11.1%)	1 141	1.05	0.75	1.47
**Illness Experience**					
No	167 (10.5%)	1 583			
Yes	100 (11.4%)	877	1.09	0.77	1.54
**Mental Health**					
Average or above	242 (11.3%)	2 136			
Below average	25 (7.7%)	324	0.65	0.37	1.15
**Relationship**					
Not stable	143 (13.1%)	1 092			
Stable	124 (9.1%)	1 368	0.66	0.47	0.93

Table 16: Question 5a (Imagine you undergo a brain health test, and it shows that you have a risk of developing brain disease. What would be your most likely reaction? - I would seek professional help (e.g., my doctor))

Group	Positive Response	n	OR	Lower CI (99%)	Upper CI (99%)
**Age**					
>= 61	12 263 (97.7%)	12 550			
<= 40	4 287 (95.6%)	4 484	0.51	0.40	0.65
41-60	9 906 (96.6%)	10 259	0.66	0.53	0.81
**Brain Disease Caregiver**					
No	14 182 (97.0%)	14 616			
Yes	12 274 (96.8%)	12 677	0.93	0.78	1.12
**Brain Research Participant**					
No	15 026 (96.9%)	15 504			
Yes	11 430 (97.0%)	11 789	1.01	0.84	1.22
**Cognitive Health**					
Average or above	24 868 (96.9%)	25 652			
Below average	1 588 (96.8%)	1 641	0.94	0.65	1.37
**Education**					
Higher	18 124 (96.7%)	18 744			
Lower	8 332 (97.5%)	8 549	1.31	1.07	1.61
**Gender**					
Woman	18 792 (96.8%)	19 413			
Other/Undisclosed	112 (91.8%)	122	0.37	0.16	0.87
Man	7 552 (97.3%)	7 758	1.21	0.98	1.50
**Healthcare Experience**					
No	16 363 (97.5%)	16 791			
Yes	10 093 (96.1%)	10 502	0.65	0.54	0.77
**Illness Experience**					
No	15 739 (96.7%)	16 271			
Yes	10 717 (97.2%)	11 022	1.19	0.98	1.43
**Mental Health**					
Average or above	23 008 (97.1%)	23 693			
Below average	3 448 (95.8%)	3 600	0.68	0.53	0.86
**Relationship**					
Not stable	11 601 (96.4%)	12 038			
Stable	14 855 (97.4%)	15 255	1.40	1.17	1.68

Table 17: Question 5b (Imagine you undergo a brain health test, and it shows that you have a risk of developing brain disease. What would be your most likely reaction? - I would seek advice from family and friends)

Group	Positive Response	n	OR	Lower CI (99%)	Upper CI (99%)
**Age**					
>= 61	6 932 (60.4%)	11 468			
<= 40	3 290 (73.6%)	84 470	1.82	1.65	2.02
41-60	6 550 (65.2%)	10 048	1.23	1.14	1.32
**Brain Disease Caregiver**					
No	8 786 (63.2%)	13 891			
Yes	7 986 (66.0%)	12 095	1.13	1.06	1.21
**Brain Research Participant**					
No	9 739 (65.7%)	14 816			
Yes	7 033 (63.0%)	11 170	0.89	0.83	0.95
**Cognitive Health**					
Average or above	15 820 (64.7%)	24 441			
Below average	952 (61.6%)	1 545	0.87	0.76	1.01
**Education**					
Higher	11 606 (64.7%)	17 946			
Lower	5 166 (64.3%)	8 040	0.98	0.91	1.06
**Gender**					
Woman	12 137 (65.5%)	18 520			
Other/Undisclosed	75 (62.5%)	120	0.88	0.54	1.43
Man	4 560 (62.1%)	7 346	0.86	0.80	0.93
**Healthcare Experience**					
No	10 248 (64.3%)	15 938			
Yes	6 524 (64.9%)	10 048	1.03	0.96	1.10
**Illness Experience**					
No	10 134 (65.3%)	15 526			
Yes	6 638 (63.5%)	10 460	0.92	0.86	0.99
**Mental Health**					
Average or above	14 660 (65.1%)	22 507			
Below average	2 112 (60.7%)	3 479	0.83	0.75	0.91
**Relationship**					
Not stable	7 449 (64.7%)	11 506			
Stable	9 323 (64.4%)	14 480	0.98	0.92	1.05

Table 18: Question 5c (Imagine you undergo a brain health test, and it shows that you have a risk of developing brain disease. What would be your most likely reaction? - I would seek information online/at the library)

Group	Positive Response	n	OR	Lower CI (99%)	Upper CI (99%)
**Age**					
>= 61	10 638 (89.5%)	11 882			
<= 40	4 029 (90.3%)	4 463	1.09	0.93	1.26
41-60	9 299 (91.6%)	10 152	1.27	1.13	1.44
**Brain Disease Caregiver**					
No	12 685 (89.6%)	14 161			
Yes	11 281 (91.4%)	12 336	1.24	1.12	1.39
**Brain Research Participant**					
No	13 666 (90.6%)	15 089			
Yes	10 300 (90.3%)	11 408	0.97	0.87	1.08
**Cognitive Health**					
Average or above	22 544 (90.5%)	24 910			
Below average	1 422 (89.6%)	1 587	0.90	0.73	1.13
**Education**					
Higher	16 883 (92.3%)	18 297			
Lower	7 083 (86.4%)	8 200	0.53	0.48	0.59
**Gender**					
Woman	17 371 (92.0%)	18 890			
Other/Undisclosed	114 (91.2%)	125	0.91	0.40	2.05
Man	6 481 (86.6%)	7 482	0.57	0.51	0.63
**Healthcare Experience**					
No	14 565 (89.6%)	16 254			
Yes	9 401 (91.8%)	10 243	1.29	1.16	1.45
**Illness Experience**					
No	14 331 (90.5%)	15 844			
Yes	9 635 (90.4%)	10 653	1.00	0.90	1.12
**Mental Health**					
Average or above	20 748 (90.3%)	22 968			
Below average	3 218 (91.2%)	3 529	1.11	0.94	1.30
**Relationship**					
Not stable	10 526 (89.8%)	11 724			
Stable	13 440 (91.0%)	14 773	1.15	1.03	1.28

Table 19: Question 5d (Imagine you undergo a brain health test, and it shows that you have a risk of developing brain disease. What would be your most likely reaction? - I would change my lifestyle if required)

Group	Positive Response	n	OR	Lower CI (99%)	Upper CI (99%)
**Age**					
>= 61	11 993 (97.7%)	12 277			
<= 40	4 364 (97.4%)	4 480	0.89	0.67	1.19
41-60	10 052 (98.0%)	10 257	1.16	0.91	1.47
**Brain Disease Caregiver**					
No	14 071 (97.5%)	14 437			
Yes	12 338 (98.1%)	12 577	1.34	1.08	1.67
**Brain Research Participant**					
No	14 981 (97.6%)	15 357			
Yes	11 428 (98.0%)	11 657	1.25	1.01	1.56
**Cognitive Health**					
Average or above	24 862 (97.9%)	25 390			
Below average	1 547 (95.3%)	1 624	0.43	0.31	0.59
**Education**					
Higher	18 148 (97.7%)	18 572			
Lower	8 261 (97.9%)	8 442	1.07	0.85	1.34
**Gender**					
Woman	18 862 (98.0%)	19 244			
Other/Undisclosed	111 (90.2%)	123	0.19	0.08	0.41
Man	7 436 (97.2%)	7 647	0.71	0.57	0.89
**Healthcare Experience**					
No	16 209 (97.7%)	16 592			
Yes	10 200 (97.9%)	10 422	1.09	0.87	1.35
**Illness Experience**					
No	15 810 (98.0%)	16 127			
Yes	10 599 (97.4%)	10 887	0.74	0.60	0.91
**Mental Health**					
Average or above	22 978 (98.0%)	23 441			
Below average	3 431 (96.0%)	3 573	0.49	0.38	0.63
**Relationship**					
Not stable	11 605 (97.3%)	11 926			
Stable	14 804 (98.1%)	15 088	1.44	1.17	1.78

Table 20: Question 5e (Imagine you undergo a brain health test, and it shows that you have a risk of developing brain disease. What would be your most likely reaction? - I would plan for the future)

Group	Positive Response	n	OR	Lower CI (99%)	Upper CI (99%)
**Age**					
>= 61	11 580 (95.3%)	12 155			
<= 40	4 159 (93.0%)	4 472	0.66	0.55	0.80
41-60	9 749 (95.6%)	10 197	1.08	0.91	1.28
**Brain Disease Caregiver**					
No	13 414 (93.8%)	14 300			
Yes	12 074 (96.4%)	12 524	1.77	1.52	2.06
**Brain Research Participant**					
No	14 362 (94.3%)	15 236			
Yes	11 126 (96.0%)	11 588	1.47	1.26	1.71
**Cognitive Health**					
Average or above	23 996 (95.2%)	25 216			
Below average	1 492 (92.8%)	1 608	0.65	0.50	0.85
**Education**					
Higher	17 680 (95.7%)	18 467			
Lower	7 808 (93.4%)	8 357	0.63	0.55	0.73
**Gender**					
Woman	18 276 (95.6%)	19 126			
Other/Undisclosed	105 (86.1%)	122	0.29	0.15	0.57
Man	7 107 (93.8%)	7 576	0.70	0.60	0.82
**Healthcare Experience**					
No	15 565 (94.6%)	16 449			
Yes	9 923 (95.6%)	10 375	1.25	1.07	1.45
**Illness Experience**					
No	15 262 (95.3%)	16 009			
Yes	10 226 (94.6%)	10 815	0.85	0.73	0.98
**Mental Health**					
Average or above	22 216 (95.5%)	23 274			
Below average	3 272 (92.2%)	3 550	0.56	0.47	0.67
**Relationship**					
Not stable	11 083 (93.5%)	11 853			
Stable	14 405 (96.2%)	14 971	1.77	1.53	2.05

Table 21: Question 6a (Brain Health Test should be affordable)

Group	Positive Response	n	OR	Lower CI (99%)	Upper CI (99%)
**Age**					
>= 61	6 138 (48.1%)	12 760			
41-60	4 846 (46.9%)	10 328	0.95	0.89	1.02
<= 40	2 532 (56.2%)	4 502	1.39	1.27	1.52
**Brain Disease Caregiver**					
No	7 225 (48.9%)	14 762			
Yes	6 291 (49.0%)	12 828	1.00	0.94	1.07
**Brain Research Participant**					
No	7 549 (48.2%)	15 671			
Yes	5 967 (50.1%)	11 919	1.08	1.01	1.15
**Cognitive Health**					
Average or above	12 696 (49.0%)	25 929			
Below average	820 (49.4%)	1 661	1.02	0.89	1.16
**Education**					
Higher	9 311 (49.2%)	18 925			
Lower	4 205 (48.5%)	8 665	0.97	0.91	1.04
**Gender**					
Woman	9 429 (48.0%)	19 626			
Man	4 014 (51.2%)	7 833	1.14	1.06	1.22
Other/Undisclosed	73 (55.7%)	131	1.36	0.86	2.15
**Healthcare Experience**					
No	8 379 (49.4%)	16 955			
Yes	5 137 (48.3%)	10 635	0.96	0.90	1.02
**Illness Experience**					
No	8 264 (50.2%)	16 451			
Yes	5 252 (47.1%)	11 139	0.88	0.83	0.94
**Mental Health**					
Average or above	11 664 (48.7%)	23 958			
Below average	1 852 (51.0%)	3 632	1.10	1.00	1.20
**Relationship**					
Not stable	6 035 (49.6%)	12 177			
Stable	7 481 (48.5%)	15 413	0.96	0.90	1.02

TABLE 22: QUESTION 6B (BRAIN HEALTH TEST SHOULD BE QUICK TO TAKE)

Group	Positive Response	n	OR	Lower CI (99%)	Upper CI (99%)
**Age**					
Age >= 61	3 871 (30.3%)	12 760			
<= 40	1 182 (26.3%)	4 502	0.82	0.74	0.90
41-60	3 221 (31.2%)	10 328	1.04	0.97	1.12
**Brain Disease Caregiver**					
No	4 434 (30.0%)	14 762			
Yes	3 840 (29.9%)	12 828	1.00	0.93	1.07
**Brain Research Participant**					
No	4 890 (31.2%)	15 671			
Yes	3 384 (28.4%)	11 919	0.87	0.82	0.94
**Cognitive Health**					
Average or above	7 847 (30.3%)	25 929			
Below average	427 (25.7%)	1 661	0.80	0.69	0.93
**Education**					
Higher	5 606 (29.6%)	18 925			
Lower	2 668 (30.8%)	8 665	1.06	0.98	1.14
**Gender**					
Woman	5 660 (28.8%)	19 626			
Other/Undisclosed	27 (20.6%)	131	0.64	0.37	1.12
Man	2 587 (33.0%)	7 833	1.22	1.13	1.31
**Healthcare Experience**					
No	5 056 (29.8%)	16 955			
Yes	3 218 (30.3%)	10 635	1.02	0.95	1.09
**Illness Experience**					
No	5 015 (30.5%)	16 451			
Yes	3 259 (29.3%)	11 139	0.94	0.88	1.01
**Mental Health**					
Average or above	7 320 (30.6%)	23 958			
Below average	954 (26.3%)	3 632	0.81	0.73	0.90
**Relationship**					
Not stable	3 441 (28.3%)	12 177			
Stable	4 833 (31.4%)	15 413	1.16	1.08	1.24

TABLE 23: QUESTION 6C (BRAIN HEALTH TEST SHOULD BE ACCURATE)

Group	Positive Response	n	OR	Lower CI (99%)	Upper CI (99%)
**Age**					
>= 61	10 826 (84.8%)	12 760			
<= 40	3 508 (77.9%)	4 502	0.63	0.56	0.71
41-60	8 542 (82.7%)	10 328	0.85	0.78	0.94
**Brain Disease Caregiver**					
No	11 992 (81.2%)	14 762			
Yes	10 884 (84.8%)	12 828	1.29	1.19	1.41
**Brain Research Participant**					
No	12 545 (80.1%)	15 671			
Yes	10 331 (86.7%)	11 919	1.62	1.49	1.77
**Cognitive Health**					
Average or above	21 529 (83.0%)	25 929			
Below average	1 347 (81.1%)	1 661	0.88	0.74	1.04
**Education**					
Higher	15 787 (83.4%)	18 925			
Lower	7 089 (81.8%)	8 665	0.89	0.82	0.98
**Gender**					
Woman	16 417 (83.6%)	19 626			
Other/Undisclosed	100 (76.3%)	131	0.63	0.37	1.07
Man	6 359 (81.2%)	7 833	0.84	0.77	0.92
**Healthcare Experience**					
No	13 943 (82.2%)	16 955			
Yes	8 933 (84.0%)	10 635	1.13	1.04	1.24
**Illness Experience**					
No	13 640 (82.9%)	16 451			
Yes	9 236 (82.9%)	11 139	1.00	0.92	1.09
**Mental Health**					
Average or above	19 919 (83.1%)	23 958			
Below average	2 957 (81.4%)	3 632	0.89	0.79	1.00
**Relationship**					
Not stable	9 913 (81.4%)	12 177			
Stable	12 963 (84.1%)	15 413	1.21	1.11	1.31

TABLE 24: QUESTION 6D (BRAIN HEALTH TEST SHOULD BE PAINLESS)

Group	Positive Response	n	OR	Lower CI (99%)	Upper CI (99%)
**Age**					
>= 61	4 009 (31.4%)	12 760			
41-60	3 727 (36.1%)	10 328	1.23	1.15	1.32
<= 40	1 967 (43.7%)	4 502	1.69	1.55	1.86
**Brain Disease Caregiver**					
No	5 453 (36.9%)	14 762			
Yes	4 250 (33.1%)	12 828	0.85	0.79	0.90
**Brain Research Participant**					
No	5 625 (35.9%)	15 671			
Yes	4 078 (34.2%)	11 919	0.93	0.87	0.99
**Cognitive Health**					
Average or above	9 205 (35.5%)	25 929			
Below average	498 (30.0%)	1 661	0.78	0.67	0.90
**Education**					
Higher	6 746 (35.6%)	18 925			
Lower	2 957 (34.1%)	8 665	0.94	0.87	1.00
**Gender**					
Woman	6 990 (35.6%)	19 626			
Other/Undisclosed	54 (41.2%)	131	1.27	0.80	2.01
Man	2 659 (33.9%)	7 833	0.93	0.86	1.00
**Healthcare Experience**					
No	6 153 (36.3%)	16 955			
Yes	3 550 (33.4%)	10 635	0.88	0.82	0.94
**Illness Experience**					
No	5 938 (36.1%)	16 451			
Yes	3 765 (33.8%)	11 139	0.90	0.85	0.97
**Mental Health**					
Average or above	8 417 (35.1%)	23 958			
Below average	1 286 (35.4%)	3 632	1.01	0.92	1.11
**Relationship**					
Not stable	4 446 (36.5%)	12 177			
Stable	5 257 (34.1%)	15 413	0.90	0.84	0.96

TABLE 25: QUESTION 6E (BRAIN HEALTH TEST SHOULD BE SUBSIDIZED BY SOCIAL SECURITY)

Group	Positive Response	n	OR	Lower CI (99%)	Upper CI (99%)
**Age**					
>= 61	5 670 (44.4%)	12 760			
41-60	4 908 (47.5%)	10 328	1.13	1.06	1.21
<= 40	2 235 (49.6%)	4 502	1.23	1.13	1.35
**Brain Disease Caregiver**					
No	7 017 (47.5%)	14 762			
Yes	5 796 (45.2%)	12 828	0.91	0.85	0.97
**Brain Research Participant**					
No	7 592 (48.4%)	15 671			
Yes	5 221 (43.8%)	11 919	0.83	0.78	0.88
**Cognitive Health**					
Average or above	11 948 (46.1%)	25 929			
Below average	865 (52.1%)	1 661	1.27	1.12	1.45
**Education**					
Higher	8 461 (44.7%)	18 925			
Lower	4 352 (50.2%)	8 665	1.25	1.17	1.33
**Gender**					
Woman	9 351 (47.6%)	19 626			
Other/Undisclosed	61 (46.6%)	131	0.96	0.61	1.51
Man	3 401 (43.4%)	7 833	0.84	0.79	0.90
**Healthcare Experience**					
No	7 762 (45.8%)	16 955			
Yes	5 051 (47.5%)	10 635	1.07	1.00	1.14
**Illness Experience**					
No	7 279 (44.2%)	16 451			
Yes	5 534 (49.7%)	11 139	1.24	1.17	1.33
**Mental Health**					
Average or above	10 977 (45.8%)	23 958			
Below average	1 836 (50.6%)	3 632	1.21	1.10	1.33
**Relationship**					
Not stable	6 127 (50.3%)	12 177			
Stable	6 686 (43.4%)	15 413	0.76	0.71	0.81

TABLE 26: QUESTION 6F (BRAIN HEALTH TEST SHOULD BE OFFERED ONLINE)

Group	Positive Response	n	OR	Lower CI (99%)	Upper CI (99%)
**Age**					
>= 61	4 084 (32.0%)	12 760			
<= 40	886 (19.7%)	4 502	0.52	0.47	0.58
41-60	2 709 (26.2%)	10 328	0.76	0.70	0.81
**Brain Disease Caregiver**					
No	4 046 (27.4%)	14 762			
Yes	3 633 (28.3%)	12 828	1.05	0.98	1.12
**Brain Research Participant**					
No	4 237 (27.0%)	15 671			
Yes	3 442 (28.9%)	11 919	1.10	1.02	1.17
**Cognitive Health**					
Average or above	7 156 (27.6%)	25 929			
Below average	523 (31.5%)	1 661	1.21	1.05	1.39
**Education**					
Higher	5 226 (27.6%)	18 925			
Lower	2 453 (28.3%)	8 665	1.04	0.96	1.12
**Gender**					
Woman	5 390 (27.5%)	19 626			
Other/Undisclosed	28 (21.4%)	131	0.72	0.41	1.25
Man	2 261 (28.9%)	7 833	1.07	0.99	1.16
**Healthcare Experience**					
No	4 814 (28.4%)	16 955			
Yes	2 865 (26.9%)	10 635	0.93	0.87	1.00
**Illness Experience**					
No	4 558 (27.7%)	16 451			
Yes	3 121 (28.0%)	11 139	1.02	0.95	1.09
**Mental Health**					
Average or above	6 769 (28.3%)	23 958			
Below average	910 (25.1%)	3 632	0.85	0.76	0.94
**Relationship**					
Not stable	3 171 (26.0%)	12 177			
Stable	4 508 (29.3%)	15 413	1.17	1.09	1.26

Table 27: Question 6g (Brain Health Test should have [other] characteristic)

Group	Positive Response	n	OR	Lower CI (99%)	Upper CI (99%)
**Age >=**					
Age >= 61	652 (5.1%)	12 760			
<= 40	229 (5.1%)	4 502	1.00	0.81	1.22
41-60	567 (5.5%)	10 328	1.08	0.93	1.26
**Brain Disease Caregiver**					
No	660 (4.5%)	14 762			
Yes	788 (6.1%)	12 828	1.40	1.22	1.61
**Brain Research Participant**					
No	756 (4.8%)	15 671			
Yes	692 (5.8%)	11 919	1.22	1.06	1.40
**Cognitive Health**					
Average or above	1 357 (5.2%)	25 929			
Below average	91 (5.5%)	1 661	1.05	0.79	1.40
**Education**					
Higher	1 145 (6.1%)	18 925			
Lower	303 (3.5%)	8 665	0.56	0.47	0.67
**Gender**					
Woman	1 036 (5.3%)	19 626			
Other/Undisclosed	9 (6.9%)	131	1.32	0.54	3.24
Man	403 (5.1%)	7 833	0.97	0.83	1.14
**Healthcare Experience**					
No	843 (5.0%)	16 955			
Yes	605 (5.7%)	10 635	1.15	1.00	1.33
**Illness Experience**					
No	772 (4.7%)	16 451			
Yes	676 (6.1%)	11 139	1.31	1.14	1.51
**Mental Health**					
Average or above	1 217 (5.1%)	23 958			
Below average	231 (6.4%)	3 632	1.27	1.05	1.54
**Relationship**					
Not stable	656 (5.4%)	12 177			
Stable	792 (5.1%)	15 413	0.95	0.83	1.09
